# The effect of ambient air pollution on birth outcomes in Norway

**DOI:** 10.1186/s12889-023-16957-1

**Published:** 2023-11-14

**Authors:** Xiaoguang Ling

**Affiliations:** Department of Economics, University of Oslo, Oslo, Norway

**Keywords:** *NO*, Ambient air pollution, Prenatal exposure, Birth outcomes

## Abstract

**Background:**

Ambient air pollution can be harmful to the fetus even in countries with relatively low levels of pollution. Most of the established literature estimates the association between air pollution and health rather than causality. In this paper, I examine the causal effects of ambient air pollution on birth outcomes in Norway.

**Methods:**

With the large sample size and geographic division of sub-postal codes in Norway, I can control for a rich set of spatio-temporal fixed effects to overcome most of the endogeneity problems caused by the choice of residential area and date of delivery. After controlling for a rich set of spatio-temporal fixed effects, my paper uses the variance in ambient air pollutant concentrations over narrow time intervals and in a small geographic area of Norway to determine how prenatal air pollution exposure affects birth outcomes. My data contain extensive information about parents as well as meteorological conditions that can be used to control for potential confounding factors.

**Results:**

I find that prenatal exposure to ambient nitric oxide in the last trimester causes significant birth weight and birth length loss under the same sub-postcode fixed effects and calendar month fixed effects, whereas other ambient air pollutants such as nitrogen dioxide and sulfur dioxide appear to be at safe levels for the fetus in Norway. In addition, the marginal adverse effect of ambient nitric oxide is larger for newborns with disadvantaged parents. Both average concentrations of nitric oxide and occasional high concentration events can adversely affect birth outcomes.

**Conclusions:**

Prenatal exposure to *NO* pollution has an adverse effect on birth outcomes. This suggests that government and researchers should pay more attention to examining *NO* pollution and that health care providers need to advise pregnant women about the risks of air pollution during pregnancy.

**Supplementary Information:**

The online version contains supplementary material available at 10.1186/s12889-023-16957-1.

## Background^1^

Ambient air pollution has become one of the major threats to human health. According to the World Health Organization [1], ambient air pollution causes millions of premature deaths each year. In addition to inducing cardiovascular and respiratory diseases such as heart attacks, strokes, and lung cancer, ambient air pollution has also been found to negatively affect the birth weight and length of newborns through prenatal exposure [2–8].^2^

Among other pollutants, *N O* is a toxic ambient air pollutant that cannot be ignored.^3^*N O* has genotoxicity [20, 21]. Chronic exposure to low concentrations of *N O* appears to induce pulmonary fibros and inhibit pulmonary defense mechanisms [22]. *N O* also has a much greater affinity for hemoglobin than oxygen [23]. Inhaled *N O* that diffuses into our blood through the alveoli and the capillaries will immdiately oxidize the Fe(II) of erythrocyte hemoglobin (Hb) to the Fe(III) state, forming methemoglobin (MetHb) [24–26], affecting the fetus through the placental barrier [27]. Based on the toxicology of *N O*, it is important to investigate whether prenatal exposure to ambient *NO* may adversely affect the health status of the newborn.

The effect of *N O* in the environment has not been thoroughly studied by the existing literature [28, 29]. As noted by the World Health Organization(WHO), “Comparisons of *N O* and *N O*_2_ are scarce and still not conclusive with regard to their relative degree of toxicity” [30]. “Although several studies have attempted to focus on the health risks of *N O*_2_, the contributing effects of these other highly correlated co-pollutants are often difficult to rule out” [31]. This is because *N O* can be rapidly oxidized to *N O*_2_ by *O*_3_.^4^

However, although both oxygen (*O*_2_) and *O*_3_ can oxidize *N O* to *N O*_2_, *O*_2_ and *N O* react very slowly in air. In the laboratory, *O*_2_ oxidizes slowly (in days) to *N O*_2_ at room temperature [35]. Since *O*_3_ at the ground level is formed mainly through photochemical reactions, when summer temperatures and solar irradiance are low, wakened photochemical reactions decrease the concentration of *O*_3_ in the environment, resulting in less *N O* being oxidized to *N O*_2_ [36]. This may be more pronounced in the winder at high latitudes (e.g., Norway) because photochemical reactions are much weaker in cold and dark winters [37, 38]. Norway’s unique high-latitude climate and natural conditions provide an opportunity to study the effect of ambient *N O*.^5^

High-quality Norwegian enrollment data allows me to consider additional confounders and mediators such as genetic pleiotropy. Birth weight loss may be the results of certain genetic defects. Parental diabetes history may affect the birth weight of the newborn [44–46]. The genotoxicity of *N O* and *N O*_2_ and the evidence of a positive association between air pollution and the risk of type II diabetes [47–49] make it worthwhile to investigate whether genetic pleiotropy is a mechanism by which ambient air pollution reduces birth weight. The established literature on air pollution and neonatal health outcomes is understudied on this issue.

This paper examines the effects of prenatal exposure to ambient air pollution in the first trimester on birth outcomes (e.g., birth weight and length) and attempts to fill gaps in knowledge about environmental *N O* that have not been well studied in the literature. With the rich registry data, I can observe the parents’ history of diabetes, which helps me overcome this problem of omitted variable bias.

## Data and inclusion and exclusion criteria

To estimate the effect of ambient air pollution on birth outcomes, data on birth outcomes and prenatal ambient air pollution exposure, i.e., the level of air pollution in the maternal residence during pregnancy, are required. Since weather has an effect on both ambient air pollutant concentrations (Additional file 1: Appendix A.3) and birth outcomes [50], meteorological conditions during pregnancy are necessary information. In addition, I need information on parental demographics, as these same parental characteristics may influence both contaminant exposure and birth outcomes. In this section, I describe how my data is constructed and how my baseline sample is selected.

### Birth outcome and parental demographic data

The birth outcome data, such as birth weight, birth length and APGAR score, is from Medical Birth Registry (MFR), a national health registry that records all births in Norway. The mother’s location in the year of delivery and the parents’ demographics are provided by Statistics Norway (SSB). The mother’s address is at the sub-postcode level, and its definition is presented in "Sub-postcode unit (*grunnkrets*) in Norway" section. The parental demographics include age, education level, nationality, immigration background, income, and wealth.^6^

Since only infants born between 2000 and 2016 can be matched to their mother’s location in my data, all newborns in my data (approximately 1 million in total) are born during this period. However, because I cannot observe ambient air pollution levels in all regions of Norway, my baseline sample contains only 46% of these newborns. In "Data interpolation and statistic description" section I will show how the baseline sample is selected from the entire population. As a means of assessing the representativeness of my sample, "Data interpolation and statistic description" section also provides a statistical description of the population and the baseline sample.

### Sub-postcode unit (*grunnkrets*) in Norway

In Norway, there is a sub-postcode geographic unit, known in Norwegian as “*grunnkrets*”, which means “basic statistical unit”. These geographic units are delineated by Statistics Norway to facilitate statistical analysis. According to Statistic Norway, *grunnkrets* are geographically cohesive and shall be as homogeneous as possible with respect to nature and economic base, communication conditions, and building structure. These small, stable geographical units can serve as a flexible basis for the presentation of regional statistics.^7^ On average, a “*grunnkrets*” is around one-third the size of a postcode zone, and the entire country is divided into more than 14,000 *grunnkrets*.^8^ In the remainder of this paper, I refer to these basic statistical units as *grunnkrets* directly.

**Fig. 1:**
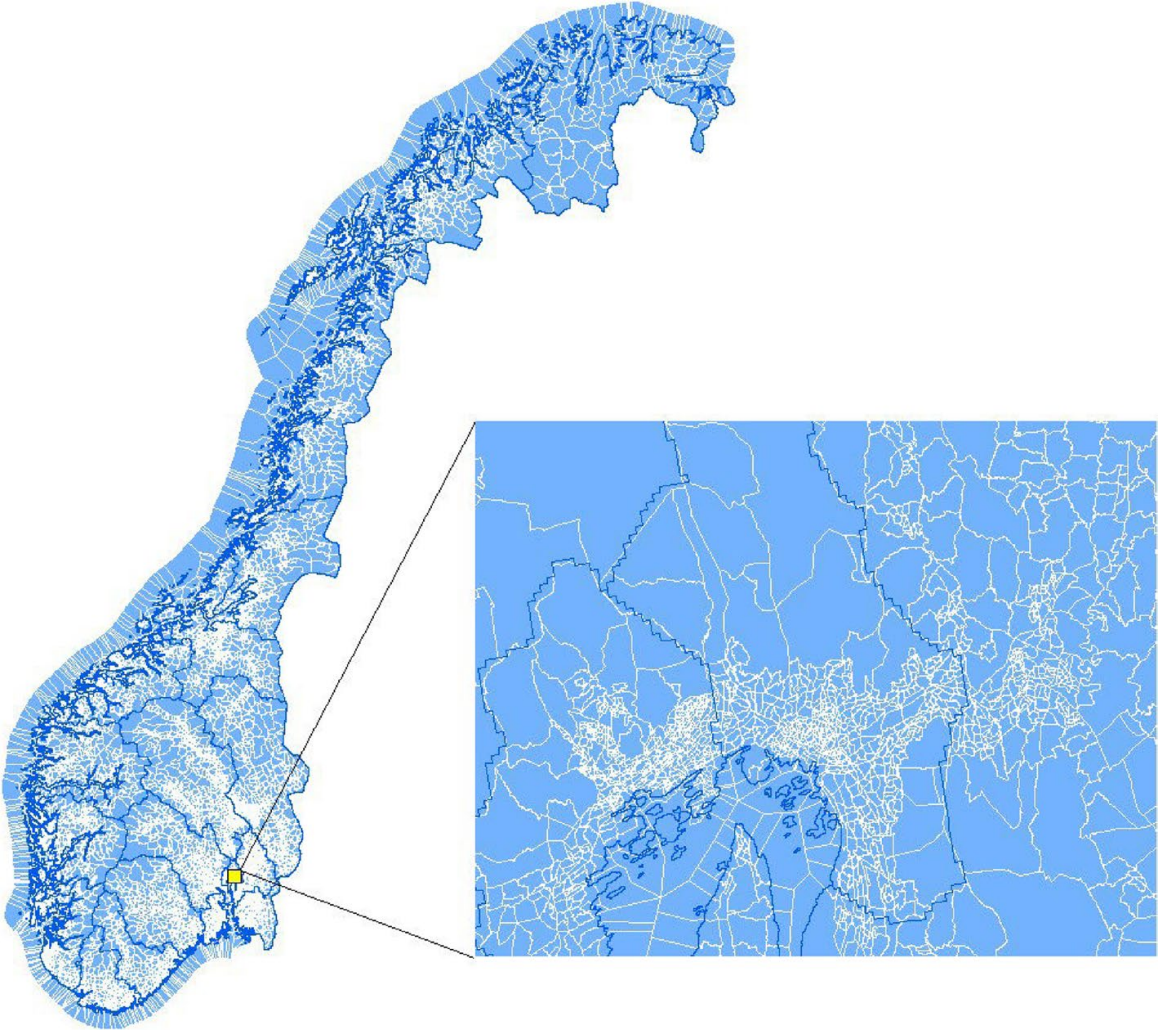
Sub-postcode unit *Grunnkrets* in Norway

Figure 1 displays a map of *grunnkrets* (small blue polygons with white outlines) in Norway. As can be seen, a *grunnkrets* is very small, and its size varies with population density.^9^ For example, in the less populated outskirts of Oslo, the capital city of Norway, *grunnkrets* are larger than in the city center (see the zoomed-out part of Fig. 1). Additional file 1: Appendix Figure D1 from the Oslo Municipality shows that there are about 50 *grunnkrets* in the Old Oslo area (part of Oslo city center and seaside), ranging in size from about 0.04 *km*^2^ to about 3 *km*^2^. The largest *grunnkrets* (number 5701) in Additional file 1: Appendix Figure D1 contains several inhabited islands.

As a geographic fixed effect, *grunnkrets* are more effective in eliminating spatial endogeneity than zip-code zones, which are commonly employed in the literature [51, 52], because they are substantially smaller in size and are intentionally designed to be internally homogeneous by Statistic Norway. Individuals may be more inclined to select where to live within a postcode zone for unobservable reasons, but moving within a *grunnkrets* is less meaningful. Compared with postcodes, it is more plausible to use infants born in the same area but at different times (and thus exposed to different levels of prenatal air pollution before birth) as counterfactuals to each other.

### Ambient air pollution data

**Fig. 2:**
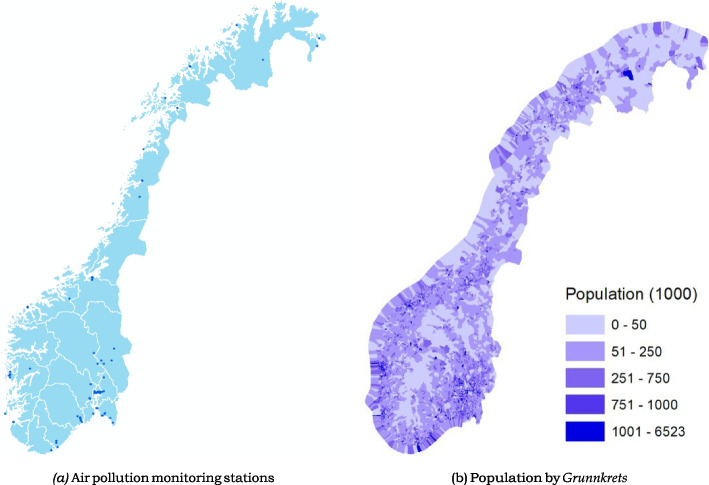
Air pollution monitoring stations are located in areas with high population density

The ambient air pollution data is provided by Norwegian Institute for Air Research (NILU), an independent, non-profit institution dedicated to the study of atmospheric composition, climate change, air quality, and environmental pollutants in Norway.^10^ During my study period, there are in total 103 ambient air pollution monitoring stations in operation or previously in operation.^11^ They are located in areas with high population density in Norway (excluding Svalbard). The location of the monitoring stations is depicted as dark blue dots in Part (a) of Fig. 2. Most of the monitoring stations are located along the Norwegian coastline, as the vast inland areas are mountainous and sparsely populated, as shown in Part (b) of Fig. 2. Due to the distribution of the monitoring stations, the values detected by the stations are mainly representative of pollution levels in urban areas. The pollution concentration data utilized in my study spans the years 1999 to 2016 to cover prenatal exposures of infants born between 2000 and 2016.^12^

The monitoring stations use a commercial Differential Optical Absorption Spectroscopy (DOAS) instrument (OPSIS AR500 analyzer) to measure the concentrations of ambient air pollutants such as *CO*, *NO*, *NO*_2_, *O*_3_, *SO*_2_, *PM*_10_, *PM*_2.5_ and *PM*_1_. The instrument performs well in detecting the aforementioned air pollutants [53].^13^ It should be noted that these monitoring stations are established (or closed) over time, and the types of pollutants that a station can detect can change over the study period. Therefore, the pollutant records generated by the monitoring stations are actually unbalanced panel data.^14^

The ambient air pollutant data provided by NILU is daily averages. I further average the data into weekly average concentrations to make it easier to construct a *grunnkrets*-time-specified panel dataset: given the large number of *grunnkrets* and the length of pregnancy (about 40 weeks in total), the *grunnkrets*-(calendar) day specified panel data is too large to process without difficulty. More importantly, my identification strategy ("Identification strategy and model specification" section) relies on the variation of air pollution in a spatio-temporal unit. In a time interval as narrow as a calendar day, the variation of air pollution (and the sample size) are not sufficient to support my identification strategy.

Table 1 depicts the weekly average ambient air pollution concentrations in Norway from 1999 to 2016. Inspired by Additional file 1: Appendix Figure A5, I divided the study period into two halves to emphasize the high *NO* concentrations prior to 2005. As can be seen from the figure, the ambient air pollution levels in Norway are much lower than in many of the areas studied in the literature. According to Additional file 1: Appendix Table D1 in the appendix, Norwegian air quality generally meets international standards. In addition, compared to air pollution levels prior to 2005, concentrations of all ambient air pollutants in Norway have decreased year by year, except *SO*_2_, indicating that the Norwegian environment has been gradually improving since the new regulations came into effect in 2002.

**Table 1:** Station level weekly average ambient air pollution in Norway between 1999 and 2016

Pollutant	1999–2004				2005–2016			
	mean	s.d	min	max	mean	s.d	min	max
*N O*	58.23	52.27	0.00	369	32.13	35.05	0.00	629
*N O*_2_	38.39	16.11	2.55	119	32.02	19.21	0.00	241
*N Ox*	126.79	91.72	0.00	671	81.18	69.96	0.00	1,178
*P M*_10_	25.81	15.43	6.56	155	20.20	11.22	0.00	135
*P M*_2.5_	13.33	5.73	3.72	59	9.51	4.88	0.72	88
*O*_3_	62.01	16.18	3.40	119	56.23	17.01	0.00	126
*SO*_2_	7.49	10.38	0.00	75	8.83	13.23	0.00	147

(1) I separate the study period into two parts to highlight the high *N O* concentration before 2005. (2) All pollutants are measured in *µg* /*m*^*3*^. (3) Here *N Ox* includes *N O*, *N O*_2_ and other nitrogen oxides. (4) The raw data provided by NILU contains negative values for the concentrations. According to NILU, negative values between -5 and 0 can be treated as 0 and those below -5 (very rare) was wrongly recorded. I thereby replaced values between -5 and 0 with 0 and treat values less than -5 as omitted

A comparison of *N O* and *N O*_2_ concentrations before and after 2005 in Table 1 shows that the average *N O*2 concentrations remained stable throughout the study period, while the average *N O* concentrations before 2005 are much higher. Surprisingly, despite the significant decrease in average ambient air pollution levels over these years, the maximum weekly concentrations of *N O* and *N O*_2_ after 2005 can still reach twice the pre-2005 levels. This suggests that extreme *N O* and *N O*_2_ pollution events continued to occur after 2005. The high volatility of weekly ambient air pollution provides the conditions for determining the effects of air pollution on birth outcomes.^15^

### Meteorological data

My meteorological information is provided by Norwegian Meteorological Institute (MET), the official weather forecasting institution that monitors Norway’s climate and conducts research. Similar to NILU, MET owns weather detection stations across the country that record meteorological information such as temperature (°*C*), air pressure (hPa), moisture (%), wind speed (m/s) and precipitation (mm). Once again, the high frequency meteorological data between 1999 and 2016 is averaged as weekly averages and will be interpolated at the *grunnkrets* level ("Data interpolation and statistic description" section).

**Fig. 3:**
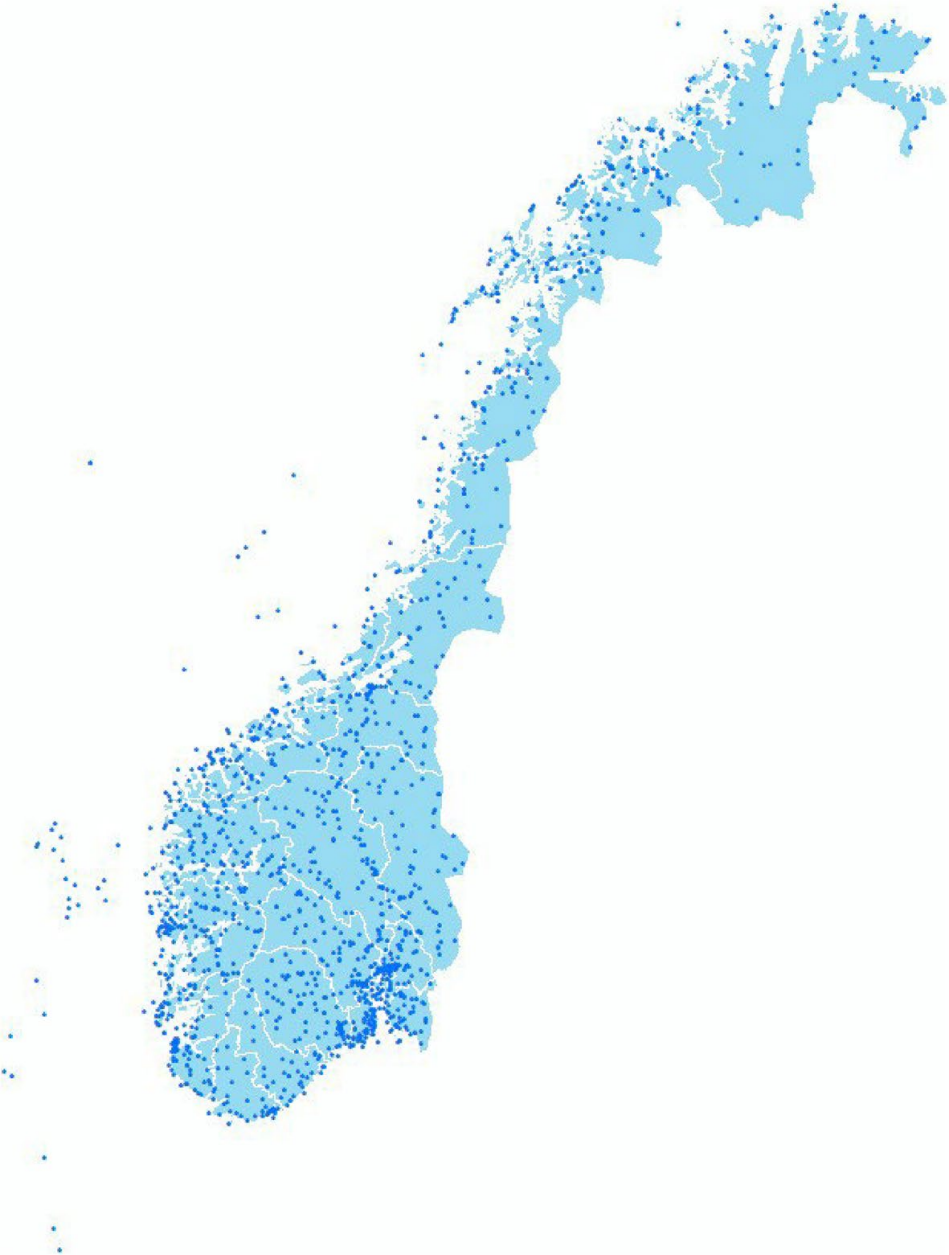
Meteorological detection stations in Norway

Figure 3 illustrates that Norway has a total of 1,198 meteorological detection stations (not including Svalbard), which is many more than the number of air pollution monitoring stations. Moreover, most of the meteorological detection stations are established early, with some of them operating more than a century ago (although not all of them are in continuous operation). As a result, the spatial resolution of the meteorological data is considerably higher and more balanced than the data from the ambient air pollution panel data.

## Data interpolation and statistic description

The above-mentioned ambient air pollution data and meteorological data are at the station level. To study the environment (*grunnkrets*) where the pregnant women lived during the pregnancy, I need to interpolate the station-level data to the *grunnkrets* level. This section describes the interpolation method and its performance. I use the same method to interpolate air pollution and meteorological conditions, but the challenge lies mainly in the interpolation of air pollution concentrations because there are not as many air pollution monitoring stations as there are meteorological monitoring stations. Therefore I focus on interpolation of air pollution concentrations in this section.

### Inverse Distance Weighting (IDW) interpolation

**Fig. 4:**
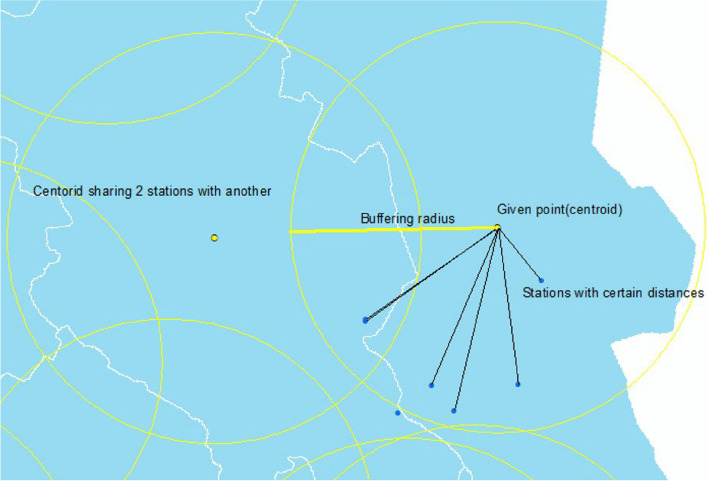
An example of the IDW interpolation method

I use the Inverse Distance Weighting (IDW) method to interpolate the station-level pollution and meteorological data to the *grunnkrets* level. As the name implies, the IDW method uses the inverse distance between *grunnkrets* and the monitoring stations to weight the station-level data. Take air pollution as an example, the IDW method uses function (1) to interpolate the ambient air pollution concentration in *grunnkrets g* at any time point *t* based on the pollution concentration detected by the monitoring stations in the neighborhood of *g* at time *t*.

1 $$Pollutio{{n}_{gt}}=\frac{\sum\limits_{i=s}^{n}{\frac{1}{d_{s}^{e}}}\times {{p}_{st}}}{\sum\limits_{i=1}^{n}{\frac{1}{d_{s}^{e}}}}$$

where *n* is the number of monitoring stations within a certain range (buffering radius) (e.g., 20 miles) around *g*. Any of these *n* monitoring stations (station *s*) records the ambient air pollution concentration value *pst* detected at time *t*. The distance between station *s* and *grunnkrets g* is *ds*. The exponent *e* is a power of the distance: the larger *p*, the higher the degree of weighting of the proximity monitoring station. In practice, I use the centroid of *grunnkrets* to represent it.^16^ Figure 4 visually illustrates the application of the IDW method on the map. The dark blue and bright yellow dots in Fig. 4 represent monitoring stations and certain *grunnkrets* centroids.^17^

The Inverse Distance Weighting method is commonly applied in the literature and performs better than many other interpolation methods such as nearest neighbor, spatial averaging, and kriging method, especially when monitoring station density is relatively low [54–56], as this is for my ambient air pollution data.^18^ The accuracy of the IDW interpolation in this paper is confirmed using cross-validation method in Additional file 1: Appendix.

The IDW method does not utilize the intrinsic characteristics of *grunnkrets*, except for the spatio-temporal association with nearby monitoring stations. In contrast, there is also a large body of literature using land use regression (LUR) interpolation methods, which utilize data on elevation, traffic, population, and vegetation cover. The rich spatial and temporal fixed effects in the regressions of "Identification strategy and model specification" section can make the IDW approach comparable to, or even superior to, the LUR approach in the sense of a partitioned regression [57]: If spatiotemporal fixed effects capture all features of the locations considered by the LUR method, then regressing the birth results on the LUR interpolated concentrations is equivalent to regressing the birth results on the IDW interpolated concentrations while controlling for spatiotemporal fixed effects. In simple terms, the latter is equivalent to the partitioned regression of the former.

Therefore, in my identification strategy, the coarseness of IDW interpolation compared to LUR interpolation depends mainly on the resolution of the controlled spatio-temporal fixed effects: when the fixed effects are at the *grunnkrets*-(calendar) month level (i.e., the *grunnkrets* and calendar-month indicators and the interaction of the two), the IDW method is not necessarily coarser than LUR interpolation because characteristics such as population and vegetation cover can be captured by fixed effects at the *grunnkrets*-(calendar) month level. Another benefit of this method is that fixed effects can also capture unobservable features of a location that are ignored in the LUR method. Of course, this benefit comes at the risk of overfitting and thus requires a large sample size.

Furthermore, even though LUR provides detailed spatial resolution, it lacks temporal resolution because the information it relies on is mostly time-invariant. In contrast, the IDW interpolation method provides good temporal resolution, but the spatial resolution is limited by the number of monitors and their separation distances [58]. It is important to note that the resolution of spatial–temporal fixed effects certainly cannot exceed the level of *grunnkrets*-(calendar) weeks, as the prenatal pollution exposure in my study is also at such a level, otherwise, prenatal ambient air pollution exposure would be perfectly multicollineary with the fixed effects.

### Data description and balance check

In this subsection, I compare my baseline sample (consisting of infants born in places where ambient air pollution can be interpolated, i.e., places with monitoring stations within 20 miles) with the rest (54%) of the population to assess the representativeness of my sample.

The map in Additional file 1: Appendix Figure D3 shows the distribution of 7,131 (out of 14,016) *grunnkrets* within 20 miles of at least one ambient air pollution monitoring station. Of these 7,131 *grunnkrets*, 91.5% are actually within 15 miles of the nearest ambient air pollution monitoring station, and 80% are even within 10 miles. The population of the 7,131 *grunnkrets* covers 67.8% of all Norwegians (data from the end of 2017). As previously mentioned in "Data and inclusion and exclusion criteria" section, each ambient air pollution monitoring station detects only certain types of pollutants. In order to simultaneously observe (or interpolate) the main pollutants, such as *N O*, *N O*_2_ and *P M*, only 5, 330 of the 7,131 *grunnkrets* could be utilized. Thus, my baseline sample represents only 46% of all newborns during the study period. Weather monitoring station coverage is not an issue here because there are so many weather monitoring stations around Norway.

Table 2 compares the characteristics of the observations covered by the interpolation (baseline sample) with those of the remaining part of the population. According to Panel A of Table 2, the newborns in my baseline sample are, on average, very similar to the rest of the population, except for birth date and weight. The infants in the baseline sample were averagely born later, as the monitoring stations are established gradually over the study period. Infants in my baseline sample are also slightly lighter, probably because there are more immigrants in my sample, as Panel B shows.

**Table 2:** Balance check by interpolated *N O* data availability

Variable	**Baseline sample**			**Population uncovered**		
	mean	s.d	Obs	mean	s.d	Obs
**A. Infantile Info**						
birth date	2009	4.42	464	2007	4.97	545
gender	0.51	0.50	464	0.51	0.50	545
weight(g)	3,492	591	464	3,521	608	544
length(cm)	50	2.71	447	50	2.69	526
APGAR1	8.7	1.2	464	8.7	1.2	544
APGAR5	9.5	0.9	464	9.4	0.9	544
**B. Parental Info**						
parity	1.59	0.74	464	1.49	0.72	539
age*m*	31	4.95	464	30	5.22	515
edu*m*	5.25	1.55	446	4.79	1.48	520
edu*f*	5.03	1.60	435	4.44	1.41	516
native*m*	0.88	0.32	464	0.90	0.30	539
native*f*	0.88	0.32	453	0.92	0.28	530
img*m*	0.70	0.46	464	0.79	0.41	539
img*f*	0.71	0.45	453	0.82	0.39	530
income*m*	234	342	449	187	124	503
income*f*	331	900	438	263	576	509
wealth*m*	484	3,411	449	166	1,353	503
wealth*f*	671	3,793	438	279	1,449	509
debt*m*	568	935	449	308	614	503
debt*f*	1,094	2,084	438	785	1,393	509
**C. Number of districts**						
municipality	118			441		
postcode	1,455			3,054		
*grunnkrets*	5,330			13,207		

(1) Obs. is the number of observations in thousand. (2) Sub-scripts “*m*” and “*f*” denote mother and father of the newborn separately. (3) *par i t y* means the number of children previously borne; Binary variable *nat i ve* indicates Norwegian nationality; *i mg* is “immigration background”, 1 for person born in Norway to Norwegian parents, 0 for other cases; *edu* is an ordered 0-8 categorical variable as defined by Statistics Norway: https://www.ssb.no/klass/klassifikasjoner/36/, e.g., edu = 4 for upper secondary education. (4) (Gross) income, wealth and debt are registered 3 years before the delivery and in thousand Norwegian kroner (NOK) at current price

Mothers in the baseline sample have children later on average; a higher proportion of parents in the sample have higher education than the remaining 54% of the population, and they are also wealthier and more likely to have an immigrant background or foreign nationality. Given that the interpolation covers most cities and more international areas, the parental characteristics in my sample are not particularly surprising. In other words, my baseline sample is more representative of the urban population in Norway.^19^ Therefore, the findings in my paper are not intended to be extrapolated to rural areas in Norway, but rather compared to other areas where ambient air pollution is at a comparable level.

## Identification strategy and model specification

As mentioned earlier, prenatal ambient air pollution exposure is non-random and associated with a large number of observable or unobservable factors, such as parental characteristics, because families can decide where to live and when to have children. A simple comparison of fetuses exposed to low and high levels of pollution during the delivery period would be subject to omitted variable bias. The ideal solution would be to randomize prenatal exposure to ambient air pollutants, but this is clearly unrealistic. My identification method attempts to mimic this hypothetical experiment by using quasi-random variations in pollution exposure across time and space. Another difficulty in identification is the measurement error induced by IDW interpolation discussed in "Data interpolation and statistic description" section.

With the National Registry data, I have sufficient power to apply rich spatio-temporal fixed effects in order to overcome both challenges to a large extent. Although I do not precisely interpolate pollution concentrations at each site, I focus only on the variation of air pollutants at a given site over a short period of time (a given month). In the case of small areas and narrow time intervals, precise self-selection of residence locations and delivery date by households is less likely to occur. Also, the abundance of temporal fixed effects improves estimation precision and compensates for the lack of accuracy of interpolation.

I use model 2 to identify the effects of air pollution on birth outcomes in order to bypass the aforementioned problems of endogeneity and coarse interpolation.

2 $${Outcome}_{j}={p}_{i}\beta +{w}_{i}{\gamma }_{j}+ {X}_{i}{\gamma }_{2}+ {g}_{i}+ {m}_{i}+{g}_{i}\times {m}_{i}+{\epsilon }_{i}$$

The dependent variables Outcome*i* in Eq. 2 are the birth weight, birth length, and APGAR scores of infant *i*. The *grunnkrets* where infant *i*’s mother lived in the year of delivery is known, and the variables *pi* and *wi* are the average interpolated concentrations of ambient air pollution and weather conditions in that *grunnkrets* prior to the mother’s delivery.^20^ The pollutants studied in my baseline regressions include *N O*, *N O*_2_, and *P M*_10_.^21^ The controlled weather conditions are humidity, precipitation, barometric pressure, temperature, and wind level. Weather conditions are important to consider because they affect both birth outcomes and air pollution, as mentioned in "Data and inclusion and exclusion criteria" section.

I retraced the pregnancy based on the birth date of the newborn. Pregnancy usually lasts about 39 weeks and is divided into three trimesters. Building on the literature, I focused on the third trimester, i.e., the 11 weeks before delivery, which is considered critical for fetal development.^22^ It is also more practical to study only the last trimester because the true gestation period may not be precisely 39 weeks. No matter how long the actual gestation period is, as long as it is longer than 11 weeks, air pollution in the 11 weeks before delivery is always what the mother is exposed to during the prenatal period.^23^ In addition, mothers are less likely to migrate during this time. By default, I assume that mothers live in the same place during the last trimester, as doctors do not recommend travel in the last weeks before delivery.^24^

The vector *Xi* represents the demographic and financial characteristics of the parents of newborn *i* listed in Panel B of Table 2. Maternal age and parity are adjusted because they themselves directly affect birth outcomes, and more experienced mothers may be more aware of the effects of air pollution and thus choose lower prenatal exposures. Parents’ economic status is adjusted, as wealthier parents may have better personal protection against air pollution, as well as better medical care and nutrition than other parents living in the same location, which resulted in better birth outcomes for their babies.

The terms *gi* and *mi* in the equation are the *grunnkrets* and calendar-month fixed effects on birth outcomes for infant *i* at birth, respectively, and *gi mi* is the interaction term of these two fixed effects. Calendar-month means the month of a particular year. For example, January 2010 and January 2011 are two different calendar-months. The calendar-month fixed effect in Eq. 2 covers both annual and seasonal time trends. The interaction term *gi mi* reflects the fact that certain spatial features have different effects on air pollution and birth outcomes at different times of a year. For example, how the topography of a place affects ambient air pollutant concentrations may depend on seasonal variations in wind direction.

In my baseline regression, there are approximately 4,000 *grunnkrets* and 200 calendar-month indicators, but not all *grunnkrets* have enough newborns in a given calendar-month to participate in regressions. Such *grunnkrets*-calendar-month combinations without sufficient samples are called singletons. After excluding these singletons, there are about 10, 000 *gi mi* combinations containing sufficient samples (about 300,000 newborn s in total). On average, in any given calendar-month, there are about 30 births per *grunnkrets*.^25^

Because the interpolated air pollution data is also at the *grunnkrets* level, I implicitly assume that infants born in the same *grunnkrets* are exposed to the same environment; after all, *grunnkrets* is both small and homogeneous within it. This is particularly evident in densely populated areas, where a *grunnkrets* can be so small as to encompass only a few blocks. Once I condition on *gi*, all spatial variations in air pollution concentrations and weather conditions are captured. Indeed, conditional on rich spatial–temporal fixed effects, the variation in prenatal exposure to ambient air pollution comes exclusively from different delivery weeks within a calendar-month.^26^

The error term *ϵi* is allowed to correlate with infants whose mothers resided in the same *grunnkrets* in the year of delivery. As a robustness check, I also allowed *ϵi* to be correlated at many different levels, including family (children of the same mother), zip-code, municipality, and the nearest monitoring station in Additional file 1: Table D3. In all these cases, the significance levels of the coefficients are very stable.

My strategy relies on the conditional independence assumption (CIA), $E[p_i\perp\epsilon_i|w_i,X_i,g_i,m_i,g_i\times m_i]$, to identify the causal effect of air pollution on birth outcomes. That is, I hypothesized that after controlling for all covariates, infants would appear to be randomly exposed to different levels of ambient air pollution. Omitted factors (confounders) that affect pollution exposure ${p}_{i}$ and birth outcomes would violate the conditional independence assumption. Thanks to the rich spatio-temporal fixed effects, it is unlikely that individuals can manipulate the time and place of delivery (i.e., prenatal exposure of the baby) in such a small spatio-temporal space; nor are shocks like improvements in urban construction (new parks, hospitals, etc.) and deterioration of living conditions (new roads in the neighborhood) likely to exist briefly in such a small spatio-temporal unit without being captured.^27^ Furthermore, because Norway has relatively little pollution compared to many developing countries, it is unlikely that there are other potential confounders, such as soil and water pollution, that happen to have the same variability as ambient air pollution.

However, it is important to note that if the choice of residence and timing of delivery are consequences of air pollution, then spatio-temporal fixed effects may be “bad controls” (i.e., covariates that are also caused by treatment) and may bias the estimated mean treatment effect. This may not be a problem because: (i) The main pollutant *N O* in my study is colorless and not very visible to the public. (ii) The average treatment effect is a weighted average of the effects estimated in the specified units in each *grunnkrets*-month. Thus, manipulations of residence and delivery time by different residents may cancel each other out. (iii) According to Additional file 1: Appendix Table D2, I find no indication of parental manipulation of delivery dates to avoid ambient air pollution.^28^

## Results

Based on regression model (2), I estimated the effect of average ambient air pollution on birth weight in the third trimester of pregnancy. Regression results are presented in Table 3. Each regression in Table 3 considers spatio-temporal fixed effects, *g*, *m* and *g m*. Other independent variables are gradually added to the regressions to test the robustness of the model specification, and column (7) of Table 3 is the baseline specification for the rest of this paper.

**Table 3:** The effect of ambient air pollution in the 3rd. trimester on birth weight

	(1)	(2)	(3)	(4)	(5)	(6)	(7)
*N O*	-0.728^∗∗^		-1.098^∗∗^	-1.361^∗∗^	-1.409^∗∗^	-1.386^∗∗^	-1.387^∗∗^
	(0.330)		(0.515)	(0.582)	(0.585)	(0.590)	(0.611)
*N O*_2_		-0.823	1.121	1.080	0.929	0.100	-0.259
		(0.804)	(1.251)	(1.600)	(1.636)	(1.697)	(1.762)
*P M*_10_					0.513	0.752	1.329
					(1.392)	(1.448)	(1.489)
parity						71.698^∗∗∗^ (2.304)	75.928^∗∗∗^ (2.428)
age*m*						0.379	-0.357
						(0.370)	(0.392)
edu*m*							8.740^∗∗∗^ (1.361)
edu*f*							6.678^∗∗∗^ (1.255)
weather	no	no	no	yes	yes	yes	yes
parental	no	no	no	no	no	yes	yes
*r* ^2^	0.441	0.441	0.441	0.435	0.435	0.456	0.464
Obs	292,349	293,526	292,343	274,334	273,112	241,913	225,239

(1) *weat her* includes humidity, precipitation, air pressure, temperature and wind; *par ent al* consists of parental economic conditions, immigration background and nationality. (2) *grunnkrets* and month fixed effect (main and interaction) are controlled for in all the regressions. (3) Cluster robust standard errors at *grunnkrets* level in parentheses, (4) Levels of significance: *** *p*<0.01, ** *p*<0.05, * *p*<0.1. (5) A radius of 20 miles and a distance power of 0.1 were used as default for pollution value interpolation. (6) Pollutants in *µg* /*m*^*3*^, birth-weight in gram

Columns (1) and (2) in Table 3 include the mean *N O* and *N O*_2_ concentrations in the third trimester, respectively, as the only independent variables to avoid potential bad controls. In both regressions, *N O* and *N O*_2_ are negatively associated with birth weight, but only the coefficient of *N O* is significant at the 5% level of significance. Including both pollutants in column (3), the sign and significance level of the coefficient of *N O* are unaffected; the coefficient of *N O*_2_ changes sign, although it remains insignificant. It appears that prenatal exposure to *N O*_2_ in the environment is not a confounder for *N O*.

As discussed in "Background" section and "Identification strategy and model specification" section, meteorological conditions affect ambient air pollutants and birth outcomes. Therefore, I control for the average meteorological conditions in the last trimester in columns (4)-(7) of Table 3. In column (5) I include the average concentration of another pollutant *P M*_10_ in the last trimester before birth. The magnitude of the coefficient on *N O* in columns (3)-(5) increases with the addition of more covariates, and remains significant at the 5% level, while the other two pollutants, *N O*_2_ and *P M*_10_, have no significant effect.^29^

In columns (6) and (7) of Table 3, I further include the parental characteristics introduced in Table 2 in regressions. As mentioned in "Data and inclusion and exclusion criteria" section, to avoid endogeneity, the parents’ financial status is registered three years before the year of birth. However, due to data limitations, the parents’ education level may be registered after the birth and thus endogenous. Therefore, I include the parents’ education separately in column (7). As expected, maternal parity and parental education level are positively associated with birth weight, but conditioning on these characteristics has no effect on the coefficient of *N O*. This supports the identification hypothesis that, given rich spatio-temporal fixed effects, prenatal air pollution exposure behaves as if it were randomly assigned to the infant. More on the manifestation of fixed effects will be discussed in the robustness checks section.

Since the coefficients on *N O* in Tables 3, 4, 5, 6 and 7 are very stable and significant at the 5% level, I conclude that a 1* µg* /*m*^3^ increase in mean environmental *N O* concentration in the third trimester reduces birth weight by approximately 1.4* g* (approximately 1/6 1/5 of the coefficient on parental education level).^30^ For each standard deviation increase (25.43* µg* /*m*^3^) in the average ambient *N O* concentration in the third trimester, birth weight decreases by 35* g*, or 1% of the average birth weight in my sample (3500* g*). The effect of *N O* found in my study is similar in magnitude to that of other pollutants studied in the literature (Additional file 1: Appendix A.1). The average concentrations of the other two pollutants *N O*_2_ and *P M*_10_ in the third trimester have no significant effect on birth weight, indicating that they are at safe concentration levels for newborns in Norway.

**Table 4:** The effect of ambient air pollution in the 3rd. trimester on birth length

	(1)	(2)	(3)	(4)	(5)	(6)	(7)
*N O*	-0.037^∗∗^		-0.044^∗^	-0.054^∗^	-0.053^∗^	-0.055^∗^	-0.052^∗^
	(0.016)		(0.025)	(0.028)	(0.028)	(0.029)	(0.029)
*N O*_2_		-0.054	0.022	0.029	0.033	0.017	-0.023
		(0.038)	(0.058)	(0.074)	(0.074)	(0.077)	(0.080)
*P M*_10_					-0.045	-0.037	-0.004
					(0.071)	(0.075)	(0.077)
parity						1.588^∗∗∗^ (0.113)	1.769^∗∗∗^ (0.119)
age*m*						0.051^∗∗∗^	0.005
						(0.018)	(0.019)
edu*m*							0.523^∗∗∗^ (0.065)
edu*f*							0.292^∗∗∗^ (0.057)
weather	No	No	No	Yes	Yes	Yes	Yes
parental	No	No	No	No	No	Yes	Yes
*r* ^2^	0.445	0.444	0.445	0.439	0.438	0.454	0.461
Obs	276,584	277,752	276,578	259,813	258,679	228,890	212,938

(1) *weat her* includes humidity, precipitation, air pressure, temperature and wind; *par ent al* consists of parental economic conditions, immigration background and nationality. (2) *grunnkrets* and month fixed effect (main and interaction) are controlled for in all the regressions. (3) Cluster robust standard errors at *grunnkrets* level in parentheses, (4) *** *p* < 0.01, ** *p* < 0.05, *p 0.1. (5) A radius of 20 miles and a distance power of 0.1 were used as default for pollution value interpolation. (6) Pollutants in *µg* /*m*^3^, birth length in millimeter

**Table 5:** Heterogeneous effect of maternal exposure to ambient air pollution in the 3rd. trimester on birth outcomes

	(1)	(2)	(3)	(4)	(5)	(6)	(7)	(8)
	boy	girl	non-ntv	native	< *i cm*^50^	> *i cm*^50^	< *i cm*^25^	> *i cm*^75^
A. Birth weight								
*N O*	-1.857^∗^ (1.084)	-1.672 (1.107)	-2.291 (1.643)	-0.893 (0.852)	-1.787^∗^ (1.022)	-1.376 (1.226)	-3.526 (2.189)	-0.856 (2.333)
*N O*_2_	-0.326 (3.068)	2.038 (3.101)	2.871 (4.277)	-1.628 (2.324)	-3.334 (2.924)	-0.476 (3.261)	-1.208 (5.792)	0.564 (6.457)
*P M*_10_	0.183 (2.742)	2.556 (2.864)	-3.310 (4.149)	2.229 (1.965)	2.326 (2.479)	5.847^∗^ (3.026)	-0.398 (4.991)	1.650 (5.479)
*r* ^2^	0.520	0.521	0.559	0.517	0.534	0.534	0.610	0.591
Obs	80,311	73,638	46,703	121,495	77,258	89,165	22,531	36,030
B. Birth length								
*N O*	-0.079 (0.051)	-0.046 (0.050)	-0.198^∗∗^ (0.082)	-0.039 (0.039)	-0.093^∗^ (0.050)	-0.062 (0.058)	-0.249^∗∗^ (0.115)	-0.061 (0.111)
*N O*_2_	0.064 (0.140)	-0.022 (0.143)	0.397^∗∗^ (0.196)	-0.128 (0.106)	-0.129 (0.148)	0.087 (0.145)	0.087 (0.351)	0.154 (0.298)
*P M*_10_	0.039 (0.124)	-0.097 (0.161)	-0.078 (0.195)	-0.005(0.093)	0.046 (0.170)	0.134 (0.139)	-0.343 (0.347)	-0.075 (0.276)
*r* ^2^	0.463	0.466	0.462	0.466	0.464	0.467	0.465	0.470
Obs	194,486	157,291	195,830	148,232	195,498	155,014	180,669	121,101

(1) Regression is based on the benchmark model. (2) “non-ntv” means at least one of the parents have immigration background or non-Norwegian nationality; “ ${i cm}_{m}^{p}$” is the maternal income at *p*th percentile. (3) The independent variables in Panel A and Panel B are birth weight and birth length separately. (4) Cluster robust standard errors at *grunnkrets* level in parentheses, (5) Levels of significance: *** *p* < 0.01, ** *p* < 0.05, * *p* < 0.1. (6) All pollutants are in *µg* /*m*^3^, birth-weight in gram, birth length in millimeter

**Table 6:** Regression by maternal exposure extent to ambient *N O* in the 3rd. trimester

*N O* conc.	< 115	< 90	< 78	< 56
A. Birth weight				
*N O*	-1.282^∗^	-1.464^∗^	-1.073	0.566
	(0.669)	(0.781)	(0.867)	(1.119)
*N O*_2_	0.175	0.843	0.258	-0.437
	(1.805)	(1.866)	(1.968)	(2.110)
*P M*_10_	1.433	1.250	1.601	1.032
	(1.497)	(1.601)	(1.661)	(1.793)
*r* ^2^	0.465	0.467	0.468	0.471
Obs	222,602	212,126	199,251	163,691
B. Birth length				
*N O*	-0.044	-0.038	-0.020	0.085
	(0.031)	(0.036)	(0.041)	(0.054)
*N O*_2_	-0.015	0.009	-0.011	-0.093
	(0.082)	(0.086)	(0.090)	(0.100)
*P M*_10_	0.001	-0.021	-0.035	-0.038
	(0.077)	(0.082)	(0.086)	(0.093)
*r* ^2^	0.462	0.464	0.465	0.469
Obs	210,501	200,909	189,049	155,822

(1) Regressions are based on the benchmark model in sub-samples with different average maternal exposure extent to ambient *N O* in the last trimester. (2) The independent variables in Panel A and Panel B are birth weight and birth length separately. (3) Cluster robust standard errors at *grunnkrets* level in parentheses. (4) Levels of significance: *** *p* < 0.01, ** *p* < 0.05, * *p* < 0.1. (5) All pollutants are in *µg* /*m*^3^, birth-weight in gram, birth length in millimeter

**Table 7:** How high-level ambient *N O* pollution events in the third trimester affect birth outcomes given last-trimester-averaged *N O* > 56 *µg*/*m*^3^

events:		99*th*^+^ percentile events			9	5*th*^+^ percentile	events	
weeks ≤ :	0	1	2	3	0	1	2	3
A. Average ambient *N O* level in trimester 3								
	69.9	73.5	76.1	76.8	63.3	65.4	69	71.8
B. Birth weight								
*N O*	-5.346^∗∗∗^	-2.723^∗∗^	-2.558^∗∗^	-1.817^∗^	-3.882	-6.317^∗^	-4.644^∗∗^	-1.691
	(1.800)	(1.387)	(1.191)	(1.073)	(5.780)	(3.324)	(2.076)	(1.504)
*N O*_2_	2.514	-0.411	-1.229	-2.777	12.134	13.994^∗^	6.678	-1.200
	(5.311)	(4.404)	(3.941)	(3.767)	(13.898)	(7.764)	(5.529)	(4.680)
*P M*_10_	0.795	0.459	0.042	-0.594	19.110	-2.425	2.417	-3.929
	(4.904)	(4.049)	(3.723)	(3.695)	(13.309)	(7.878)	(5.551)	(4.521)
*r* ^2^	0.477	0.471	0.468	0.467	0.486	0.486	0.481	0.477
Obs	36,588	49,596	54,373	55,182	8,834	20,235	35,268	44,776
C. Birth length								
*N O*	-0.253^∗∗∗^	-0.142^∗∗^	-0.140^∗∗∗^	-0.117^∗∗^	0.090	-0.180	-0.151	-0.104
	(0.085)	(0.064)	(0.054)	(0.051)	(0.260)	(0.156)	(0.098)	(0.071)
*N O*_2_	0.308	0.130	0.085	0.012	0.198	0.735^∗∗^	0.302	0.180
	(0.237)	(0.194)	(0.173)	(0.168)	(0.651)	(0.366)	(0.252)	(0.205)
*P M*_10_	0.001	0.049	0.092	0.063	0.207	-0.376	0.132	-0.100
	(0.232)	(0.194)	(0.172)	(0.172)	(0.585)	(0.359)	(0.251)	(0.214)
*r* ^2^	0.464	0.462	0.459	0.459	0.481	0.481	0.471	0.468
Obs	33,968	45,907	50,340	51,054	8,201	18,770	32,685	41,450

(1) Regression is based on the benchmark model in observations whose average maternal *N O* exposure in the last trimester is greater than the average (56* µg* /*m*^3^). These observations are further classified in to sub-samples according to the number of “high-level *N O* pollution events”, which is defined as weeks with average ambient *N O* concentration higher than 99*th*/95*th* percentile (170 µg /*m*^3^ and 110* µg* /*m*^3^ separately) of the weekly *N O* concentration in the last trimester. (2) The average ambient *N O* level in the last trimester for each sub-group is in Panel A. The independent variables in Panel B and Panel C are birth weight and birth length separately. (3) Cluster robust standard errors at *grunnkrets* level in parentheses, (4) *** *p* < 0.01, ** *p* < 0.05, * *p* < 0.1. (5) All pollutants are in *µg* /*m*^3^, birth-weight in gram, birth length in millimeter

Based on these findings, *N O* may pose a greater threat to newborns in Norway than other ambient air pollutants, especially in large cities such as Oslo and Bergen. In recent years, the quarterly average ambient *N O* values in Norway have typically been 60* µg* /*m*^3^ in winter. If the adverse effect of environmental *N O* pollution on birth weight is linear, then winter *N O* pollution may contribute to a birth weight loss of 84* g* for this group of infants on average, or 2.4% of the average birth weight in Norway. In Bergen, Norway’s second largest city, monthly *N O* pollution levels can be as high as 120* µg* /*m*^3^ (2019) and even reach 275* µg* /*m*^3^ (2010) in some months in “Danmarksplass” (around the city center), which may cause even more birth weight loss.

The effect of air pollution on birth length has similar patterns, as shown in Table 4. In columns (4)-(7) of Table 4, the coefficient of *N O* is stable, hovering around 0.052* mm*. Although the coefficients of all three pollutants are insignificant at the 5% level, the coefficient of *N O* is significant at the 10% level, while the coefficients of the other two pollutants are far from significant (*t*-statistic 0.5). The coefficients on N O indicate that during the third trimester of pregnancy, every 1 µg /*m*^3^ increase in the ambient *N O* concentration results in a birth length reduction of 0.052* mm* (about 1/10 1/6 of the coefficients on parental education level). One standard deviation increase of ambient *N O* concentration in the third trimester would reduce birth length by 1.4* mm*, which is 0.3% of the mean birth length (500* mm*) in the baseline sample. This effect is comparable to the association between *N O*_2_ and birth length found in the literature (Additional file 1: Appendix A.1).

I also examined the effect of ambient air pollution during the last three trimesters on infant APGAR1 and APGAR5 scores, but did not find any significant effects (results not shown). This may be due to the small variation in APGAR scores in Norway, which is described in "Birth outcome and parental demographic data" section. In conclusion, I find that prenatal exposure to environmental *N O* in the third trimester reduced birth weight and birth length, whereas prenatal exposure to ambient *N O*_2_ and *P M*_10_ are at safe levels for Norwegian newborns.

In Additional file 1: Appendix Section C, I first evaluate the sensitivity of my identification strategy to IDW interpolation, which affects both estimation and statistical inference (as it affects sample size). I then indirectly test the conditional independence assumptions underlying my identification strategy by testing for spatio-temporal fixed effects and other potential confounders. Finally, I discuss the case of mothers moving pre/post-natally, which may lead to measurement error and make spatial fixed effects a “bad control” [59].

## Heterogeneity

This section examines the heterogeneous effects of prenatal exposure to ambient air pollution on birth outcomes across subgroups categorized by demographics and ambient air pollution levels. It is important to note that splitting the sample into subgroups reduces the number of observations within a *grunnkrets*-month. As a result, more singletons are excluded from the regression, and the precision of the estimates is expected to be reduced.

### Heterogeneity by demographics

Table 5 report regressions of subgroups with different demographic characteristics. The first two columns in Table 5 are regressions by gender grouping of infants. Consistent with the literature, the *N O* effects in column (1) are greater and more significant than those in column (1), implying that male newborns appear to be more susceptible to prenatal ambient air pollution than female newborns.

In columns (3) and (4) of Table 5, I split the sample into two groups based on immigrant background and nationality, where “non-ntv” (non-native) is defined as having at least one parent with an immigrant background or with non- Norwegian nationality, while “native” means that both parents are Norwegian and have no history of immigration. Comparing the results in columns (3) and (4) with the baseline regression, we can see that environmental *N O* concentrations have a greater marginal adverse effect (2-4 times) on birth outcomes for non-native infants. In contrast, for native infants, the marginal effect of maternal *N O* exposure is smaller and less pronounced than in the baseline regression. One possible explanation is that immigrants are more exposed to ambient air pollution than natives due to their occupation and the effect of air pollutants on birth outcomes is non-linear (marginal increment).^31^

I further examine the heterogeneity of the effect of air pollution in terms of mothers’ income in columns (5)-(8) of Table 5.^32^ The annual after-tax income of mothers in columns (5) and (6) is below and above the mean (${icm}_{m}^{50}$), respectively. It can be seen that infants whose mothers have below-average income appear to be more vulnerable to prenatal *N O* exposure than those who are financially well off. To highlight this heterogeneity , I further compared newborns whose mothers’ income is below the first quartile ($<icm_m^{25}$ , the worse off) and above the third quartile ($>icm_m^{75}$, the better off). Not surprisingly, the marginal adverse effect *N O* on birth weight is much larger and more significant in poorer conditioned infants. This result is consistent with the findings for immigrants in columns (3)-(4).

In summary, I find that prenatal exposure to ambient *N O* is more detrimental to male than female infants. Also, the marginal adverse effects of ambient *N O* on fetuses are larger and more significant for families with immigrant background/nationality and/or lower incomes.

### Heterogeneity by prenatal exposure level

The effects of ambient air pollutants on birth outcomes may be nonlinear. For example, below certain safe levels, even long-term prenatal exposure may not affect birth outcomes. On the contrary, at high pollution levels, short-term exposures may also cause serious harm. In this subsection, I first examine the response of birth outcomes to air pollutant concentrations in infants exposed to different average levels of air pollution during the last trimester. Note that there are 11 weeks in the last trimester, the high average trimester prenatal exposure levels may come from very high air pollution in just a few weeks (while the other weeks have very low pollution levels. Additional file 1: Appendix Figure D5 illustrates this scenario). Therefore, I studied further to see if the adverse health effects on birth outcomes were driven by these occasional high levels of air pollution events.^33^

Columns (1)-(4) of Table 6 are regressions for sub-samples with mean prenatal *N O* exposure levels below the 99*th*, 90*th*, 75*th*, and 50*th* percentiles in the last trimester. It appears that the exclusion of observations with the highest prenatal *N O* exposure does not change the coefficient of *N O* much, especially for birth weight. When prenatal exposure is below average (column (4)), the *N O* coefficients in panels A and B are no longer negative, indicating that the last trimester of below-average prenatal *N O* exposure is safe. The other two pollutants, *N O*_2_ and *P M*_10_, have no significant effect on birth outcomes. Also, according to Table 6, the marginal effect of last-trimester ambient *N O* pollution on birth outcomes appears to be greater when the average *NO* concentration level is higher.

For observations with above-average prenatal last-trimester *NO* exposure (56* µg* /*m*^3^), I further divided them into groups based on the number of “high-concentration environmental *N O* events” they experienced in the last trimester. Here a “high ambient *N O* event” is defined as a week in which the ambient *N O* concentration is above certain percentiles, such as the 99*th* (170* µg* /*m*^3^) and 95*th* (110* µg* /*m*^3^) percentiles. The regression results, as well as the mean prenatal *N O* exposure in the last trimester corresponding to each subgroup, are shown in Table 7. Taking columns (1) and (4) of the table as examples, the newborns in both columns are exposed to above-average levels of ambient *N O* in the last trimester prenatally, but only the infants in column (4) experienced “high-level ambient *N O* events” (3 weeks), whereas the infants in column (1) are not exposed to any high levels of ambient *N O* pollution events.

Interestingly, columns (1)-(4) of Table 7 suggest that the more “ambient *N O* concentrations above the 99*th* percentile event” in the sample with above-average prenatal *N O* exposure in the last trimester, the smaller the marginal effect of *N O*, although the average *N O* in each subgroup concentration increases from column (1) to column (4). When there is no such “high ambient *N O* event” in the last trimester (column (1)), the marginal effect of *N O* is four times greater than in the baseline regression. This suggests that chronic exposure to relatively high levels of ambient *N O* pollution (column (1)) is more detrimental than occasional high levels of ambient *N O* pollution events for fetuses whose mothers are exposed to above-average levels of ambient *N O* pollution in the last trimester. The same pattern is shown in columns (6)-(8) of Table 7. In these columns, “high ambient *N O* events” are defined as weeks when the weekly average ambient *N O* concentration is above the 95*th* percentile. The estimate in column (5) is very imprecise because there are too few observations.

Similarly, for observations with below 78* µg* /*m*^3^ (75th percentile) prenatal *N O* exposure, I grouped them into four groups based only on the number of *N O* pollution events above the 95*th* percentile concentration in the last trimester.,^34^,^35^ The regression results for each subgroup for the last three months of prenatal *N O* exposure are presented in Table 8. As seen in Panel A, the mean ambient *N O* concentrations are low for all four subgroups. Compared to Table 7, the sign of the coefficient on *N O* is insignificant and can even be positive when there are fewer than three high-level *N O* pollution events (columns (1)-(3)), whereas when the number of high-level *N O* pollution events increases to three, the magnitude of the adverse effect becomes much larger than in the baseline regression and is significant at the 5% level. It appears that for those with very low prenatal *N O* exposure, occasional environmental *N O* concentration events “above the 95*th* percentile” in the last trimester also adversely affect their birth weight and length.

**Table 8:** How *N O* 95*th*^+^ percentile events in the third trimester affect birth outcomes given last-trimester-averaged *N O* < 78* µg* /*m*^3^

weeks ≤	0	1	2	3
A. Average ambient *N O* level in trimester 3				
	30.1	33	34.6	35.1
B. Birth weight				
*N O*	-0.869	5.724	0.894	-4.566^∗∗^
	(0.906)	(13.204)	(1.227)	(2.213)
*N O*_2_	0.293	9.550	-0.450	3.297
	(1.976)	(38.983)	(2.120)	(7.254)
*P M*_10_	1.412	-24.531	0.577	9.481
	(1.668)	(38.419)	(1.766)	(6.676)
*r* ^2^	0.468	0.552	0.468	0.498
Obs	195,790	2,673	165,227	29,473
C. Birth length				
*N O*	-0.009	0.509	0.105^∗^	-0.276^∗∗∗^
	(0.043)	(0.649)	(0.058)	(0.105)
*N O*_2_	-0.004	-0.488	-0.056	0.331
	(0.091)	(1.886)	(0.101)	(0.319)
*P M*_10_	-0.042	-1.580	-0.089	0.185
	(0.086)	(1.753)	(0.094)	(0.301)
*r* ^2^	0.464	0.597	0.466	0.492
Obs	185,866	2,445	156,990	27,671

(1) Regression is based on the benchmark model in observations whose average maternal *N O* exposure in the last trimester is less than the 78* µg* /*m*^3^. These observations are further classified in to sub-samples according to the number of “high-level *N O* pollution events”, which is defined as weeks with average ambient *N O* concentration higher than 99*th*/95*th* percentile (170* µg* /*m*^3^ and 110 *µg* /*m*^3^ separately) of the weekly *N O* concentration in the last trimester. (2) The average ambient *N O* level in the last trimester for each sub-group is in Panel A. The independent variables in Panel B and Panel C are birth weight and birth length separately. (3) Cluster robust standard errors at *grunnkrets* level in parentheses, (4) *** *p* < 0.01, ** *p* < 0.05, * *p* < 0.1. (5) All pollutants are in *µg* /*m*^3^, birth-weight in gram, birth length in millimeter

By combining Tables 7 and 8, I conclude that for the sample with above-average prenatal environmental *N O* exposure levels in the last trimester, long-term exposure to relatively high ambient *N O* levels caused more harm than occasional high ambient *NO* events, whereas for observations with relatively low average last trimester prenatal environmental *N O* exposure levels, occasional high ambient *N O* events, if present, are also harmful to birth outcomes.

## Conclusion

In this paper, by using the variance in prenatal ambient air pollution exposure levels among infants born within a specific calendar-month in the same sub-zip-code area, I find that exposure to ambient nitric oxide (*N O*) in the last trimester of pregnancy can significantly reduce birth weight and length in Norwegian children born between 2000 and 2016. On average, each standard deviation increase (25.4* µg* /*m*^3^) in prenatal exposure to *N O* resulted in a 1% decrease in birth weight and a 0.3% decrease in birth length, which is similar to the effects of other studies on the effects of ambient air pollutants. Pollution levels of other types of ambient air pollutants, such as *N O*_2_, *P M*_10_ and *O*_3_, appear to be safe for Norwegian fetuses. Prenatal exposure to *SO*_2_ in the environment appears to have a negative effect on birth weight and length, but there are not enough observations to make a precise estimate. I do not find an effect of prenatal ambient air pollution exposure on APGAR scores.

The affinity of *N O* for hemoglobin may be a contributor to this adverse effect. The diffusion of inhaled *N O* into the blood of the pregnant woman through the alveoli and capillaries oxidizes the Fe(II) of red blood cell hemoglobin (Hb) to the Fe(III) state, forming methemoglobin (MetHb) and impairing oxygen transport. As a result, the fetus is exposed to methemoglobin through the placental barrier. Although ambient air pollution is associated with diabetes, which in turn affects birth weight, I have not found any evidence that ambient *N O* has such a mechanism.

It would be interesting to further confirm the mechanisms by which environmental *N O* pollution affects the fetus. Though the literature has found a link between birth outcomes and long-term health outcomes, it is not clear whether reduced birth weight and length due to ambient air pollution can also affect long-term health outcomes. Understanding the mechanisms by which pollutants affect birth outcomes can help assess their long-term effects.

In addition, I find that both average ambient *N O* in the last trimester and occasional high ambient *N O* pollution events can be harmful to the fetus. Although ambient air quality in Norway is generally high and has been improving in recent years, there are weeks with high ambient *N O* concentrations that are harmful to the last trimester fetus. As found in the literature, reductions in birth weight and length may have a negative impact on the long-term health status of children. This poses a challenge to environmental pollution management and policy development: not only to focus on average pollutant levels, but also to pay attention to the containment of short-term high pollution events.

Prenatal exposure to ambient *N O* also has heterogeneous effects on different groups. Consistent with the literature, I find that male infants are more susceptible to environmental *N O* pollution than female infants. Infants from economically and/or ethnically disadvantaged families are more affected than children from better-off families. This is not surprising because most immigrants live in large cities like Oslo, where ambient *N O* concentrations are occasionally quite high during some weeks, and as mentioned earlier, the marginal effect of ambient *N O* on birth outcomes is greater when air pollution levels are high. Another possible explanation is that less privileged mothers are physically more vulnerable to the effects of ambient air pollution. Due to the nature of their work, they may also engage in more outdoor activities. Future studies may examine why newborns of poorly conditioned parents are more vulnerable to ambient air pollution and how to protect them. If infants’ long-term health is made worse by prenatal exposure, and thus disadvantaged in the labor market in the future, they may be more likely to be exposed to the same harmful environment—parental and offspring air pollution exposure reinforcing each other and create a poor-health (and also poverty) trap.

## Limitation

A limitation of the study methodology in this paper is that the addresses of pregnant women are updated annually, and I may not have been able to accurately determine where the mothers resided during pregnancy. This may be the reason why I find no significant effect of prenatal exposure to ambient air pollution in the first two months, although the literature suggests that most abnormal fetal development occurs in the last trimester. Using the mother’s workplace address in the year of birth, which is regularly recorded by social welfare and tax agencies, may be a solution for the future. Another limitation of the model used in this paper is that although it controlled for a rich set of spatio-temporal fixed effects, there may potential confounders omitted, such as traffic noise levels.

### Supplementary Information

**Additional file 1.**

## Data Availability

The data used in this study are available from the following sources: (1) Geo data (https://kartkatalog.geonorge.no), weather data (https://www.met.no/en) and pollution data (https://api.nilu.no) are publically available via the links above; (2) Demographic data and birth outcome data are from Statistics Norway (https://www.ssb.no/en), which requires special application to access. The author can provide guidance on applying for these data.

## Footnotes

1 A more detailed background is provided in Additional file 1: Appendix A.

2 Birth outcomes such as birth weight and birth length are strong indicators of fetal and neonatal mortality as well as a variety of other long-term health outcomes [9–13]. Low birth weight can also result in high economic costs for families and society [14]. In addition, the adverse effects of air pollution on birth outcomes may be heterogeneous. For example, boys are more affected by ambient air pollution than girls [15]. Meanwhile, the advantaged may also suffer higher exposure to ambient air pollution due to higher rates of participation in outdoor sports [16].

3 The toxicology of *N O* is complex. At very low levels, *N O* plays a key role in our cardiovascular, neurological, and immune systems [17]. Low doses of inhaled *N O* therapy is often used as an effective vasodilator in the treatment of certain respiratory diseases. The safe dose of inhaled *N O* (i*N O*) therapy in neonates has not been fully established, but most studies start with a dose of 25 µg /*m*^3^ and gradually decrease the dose. A does of 50 µg /*m*^3^ has been used in adults. See also: [18] and [19]. In my study, the ambient *N O* concentration can be much higher than such a level.

4 There are two studies finding that the increase in ambient *N O* exposure during pregnancy is associated with a higher risk of low birth weight [32] and childhood acute lymphoblastic leukemia [33]. There are also a few studies on the relationship between *N O* and diseases such as asthma [34].

5 The annual average ambient *N O* concentrations in Norway are higher than *N O*_2_, which is different from most of other European and American areas studied in the existing literature  [39–42]. In addition, the average monthly temperature in Norwayis below 10 °C for 8 months of the year. Even in summer, the maximum monthly average temperature stays below 21 °C. Although *N O*_2_ exists in gaseous form in air under normal ambient conditions due to its low partial pressure in the atmosphere (908*mmH g* at 25 °C) [43], the gas will be compressed and much heavier than air if enough *N O*_2_ molecules are present in the ambient air.

6 Because financial status may be affected by family planning and therefore endogenous, I use information on income, wealth, and debt registered in the two years prior to the birth. The same endogeneity may be true for parents’ education levels. However, since annual education registration data are not available, I use the highest education level registered in the dataset. Fortunately, when I restrict the sample to observations where the parents’ education level is registered at least two years prior to the birth, the results remain the same (results not shown), meaning that there is unlikely to be an endogeneity problem.

7 The definition of *grunnkrets* is available on Statistic Norway’s webpage: https://www.ssb.no/a/metadata/conceptvariable/vardok/135/en

8 My baseline sample consists of 5,330 *grunnkrets*, covering 38.6% of Norway’s area (118 municipalities and 1,455 postcode zones) and 46% of the national population. As part of the robustness check, the regression in Additional file 1: Table C1 contains as many as 7853 *grunnkrets* (56.8% of the country’s area).

9 *grunnkrets* are also very small in terms of population. By the end of 2016, there are on average 549 people per *grunnkrets* in my baseline sample. The first three quartiles of the population per *grunnkrets* are 196,400 and 732. In fact, 99% of *grunnkrets* have fewer than 2,435 population, and these *grunnkrets* cover a total of 97% of country’s population. This suggests that most people reside in small *grunnkrets* (i.e., as shown in Fig. 1, the more densely populated the area, the smaller the *grunnkrets*). Notably, the population of the most populous *grunnkrets* in my sample increases from 3,455 in 1999 to 6,052 in 2016, while the average population per *grunnkrets* only increases from 444 to 549 over the same period. This indicates that the population growth is uneven across *grunnkrets*. The Norwegian population has gradually become more concentrated.

10 The data collected by NILU is also an important source of data for Norwegian Environment Agency.

11 Examples of the monitoring stations: https://www.nilu.com/facility/nilus-observatories-and-monitoring-stations/.

12 In "Data interpolation and statistic description" section, I explain how I interpolate the air pollution concentrations at the *grunnkrets* level based on the station-level panel data.

13 *N O* (*O*_3_) values detected by DOAS may be lower (higher) than those detected by various traditional point sampling techniques. This may be caused by the steep vertical gradient of the *N O O*_3_ *NO*_2_ system (the *N O O*_3_ *NO*_2_ reaction rapidly changes the components of the air after *N O*_*2*_ is released). If this is the case, the value detected by DOAS should be closer to the actual pollutant concentration [53].

14 The detection of *CO* and *P M*_1_ starts quite late and only covers a very small fraction of my sample. I therefore exclude the two pollutants from my study. Omitting the two ambient air pollutants should not affect my estimation, since the ambient *CO* concentration is very low in Norway. As a type of *P M* (particulate matters), *P M*_1_ is highly correlated with other *P M* such as *P M*_10_, which is has been detected for many years.

15 Additional file 1: Appendix Figure D2 shows the percentage of weeks with high levels of *N O* (95th percentile, or 110* µg* /*m*^3^) at the monitoring station level between 1999 and 2016. Unsurprisingly, high levels of ambient *N O* are common in urban areas. Weekly data collected at a number of monitoring stations in major cities such as Trondheim (middle bubble), Bergen, and Stavanger (two large bubbles on the west coast) show that *N O* concentrations exceed 110* µg* /*m*^3^3for about 15% of weeks. In Oslo, the capital of Norway (yellow bubble), *N O* concentrations exceeded 110* µg* /*m*^3^ in 32% of weeks between 1999 and 2016.

16 Note that there may be some textitgrunnkrets that do not have monitoring stations nearby, in which case I cannot interpolate the air pollution levels for these textitgrunnkrets. This is why my baseline sample does not cover the entire population. For *grunnkrets* with only one nearby monitoring station, the interpolated concentrations are exactly the same as for the nearby (only) monitoring station.

17 The map in Fig. 4 is for illustrative purposes only. In fact, because *grunnkrets* are very small and most air pollution monitoring stations are located in large cities, most *grunnkrets* within these cities actually share the same monitoring stations if a 20-mile radius is used. In contrast, for many *grunnkrets* in rural areas, there are no monitoring stations nearby at all.

18 Note that ambient air pollution spreads in the ground atmosphere after emission, and the location of the monitoring station is not the source of the pollution. Therefore, I cannot use data from monitoring stations alone to model the dispersion of pollutants in the air. Instead, data from the monitoring stations are used to represent the exposure to pollution of residents living near the monitoring stations.

19 Even so, as described in "Background" section, ambient air pollution levels in these urban areas are still low overall compared to air pollution levels in other countries, according to air quality guidelines.

20 Note that once the date of birth of baby *i* is given, prenatal exposure to ambient air pollution is also known. Since birth outcomes are “one-time” rather than recurrent, i.e., there is no temporal variation in birth outcomes for individual *i*, my sample is actually pooled cross-sectional data rather than panel data. Therefore, I can omit the time subscripts in Eq. 2.

21 The analysis of other pollutants such as *P M*_2.5_, *SO*_2_ and *O*_3_ is included in the robustness test section (Additional file 1: Appendix section C) because there are fewer monitoring stations for these three pollutants and the samples are smaller.

22 I studied the average ambient air pollution and weather conditions for all three quarters in Additional file 1: Appendix Table D4 and found that only the last trimester has significant significant effects.

23 In extreme cases, pregnancy may even be shorter than 11 weeks, and the weight of the stillbirth is also registered. I may thus wrongly specify the prenatal pollution exposure levels, but these cases are very rare.

24 In the robustness check section, I will consider mothers moving between *grunnkrets*.

25 In the robustness check section, I apply more coarse spatio-temporal fixed effects, such as postal-code-(calendar) month levels, which cover more regions with smaller populations.

26 The graph in Additional file 1: Appendix Figure D4 gives an example: Two infants were born in the same calendar quarter, but have different prenatal exposures to ambient *N O* simply because they were born in different weeks of the calendar-quarter. The large amount of registry data provides me with sufficient power to use differences in prenatal exposure within a calendar-month to determine its impact on birth outcomes.

27 In robustness tests, I will show that, conditional on spatio-temporal fixed effects, prenatal air pollution exposure is effectively like a random assignment of the parental characteristics *Xi* mentioned above to infants. That is, once spatio-temporal fixed effects are controlled for, there is no need to use covariates *Xi* in the Eq. 2 to identify the effect of ambient air pollution on birth outcomes.

28 I analyzed the characteristics of families who chose to give birth in different seasons and also did not find meaningful indigenous differences (results not shown). In the robustness testing section, I also discuss more about mothers moving in the year before delivery, which may be an indication of choice of residence.

29 I control for the other types of pollutants in the robustness check section and find that the inclusion of these additional controls has little effect on the coefficient of *N O*.

30 *edu* is an ordered categorical variable taking values from 0 to 8, where 0 indicates no education and 8 indicates postgraduate education, as defined by SSB: https://www.ssb.no/klass/klassifikasjoner/36/. One additional one unit increase in *edu* can be interpreted as an increase in education level, which is arguably more important than an additional year of education.

31 For example, 12.6% of immigrants in Norway are in primary occupations, compared to 2.7% of native Norwegians. Data from Statistics Norway: https://www.ssb.no/en/arbeid-og-lonn/sysselsetting/statistikk/sysselsetting-blant-innvandrere-registerbasert

32 I also classify the sample according to the father’s income, both parents’ income and wealth, all with relatively similar results (not shown). The reason for using mother’s income by default is that more observations register information about the mother.

33 I similarly examine the effects of occasional high level air pollution events of *N O*_2_ and *P M*_10_ on birth outcomes in Additional file 1: Table D5 and find that these events do not affect birth outcomes.

34 I also tried to use observations with below-average prenatal *N O* exposure (56* µg* /*m*^3^) in the last trimester, as in Table 8, but “high ambient *N O* events” are too few in these observations and the estimated noise is too large.

35 Events above the 99*th* percentile are not studied here because they are extremely rare and the sample size is not sufficient for regression.
