# Supplementary material for: The effect of ambient air pollution on birth outcomes in Norway
Source: BMC Public Health. 2023 Nov 14;23:2248. doi: 10.1186/s12889-023-16957-1 (PMC10647155; doi:10.1186/s12889-023-16957-1)
Supplement: Supplementary file 1 — Additional file 1. [file 12889_2023_16957_MOESM1_ESM.docx]

# **Appendices**

1. **Literature review**
   1. **Ambient air pollution and birth outcomes**

A large body of literature suggests that prenatal exposure to air pollutants, such as carbon monoxide (*CO*), nitrogen oxides (*N Ox* ), ozone (*O*3), sulfur oxides (*SOX* ), and particulate matter (*P M* ), during the prenatal period, especially in the last trimester, is associated with poor birth outcomes.[36](#_bookmark1) [3] find that an inter-quartile increase in prenatal exposure to *N O*2, *CO* and *P M* is associated with a 9 16*g* reduction in birth weight.[37](#_bookmark2) [4] also find similar effects for *P M* : a 10 *µg* /*m*3 increase in daily *P M*10 (*P M* of 10 microns or less in diameter) concentration in the third trimester is correlated with an 11*g* reduction in birth weight. Ambient pollutants such as *O*3 [51] and *SO*2 [5, 6] are also associated with low birth weight. Aside from birth weight, ambient air pollution also reduces birth length and fetal head circumference. Each 1*µg* /*m*3 increase in *N O*2 and *P M* reduces fetal head circumference by 0.12 0.18*mm* [7]. A 10*µg* /*m*3 increase in *N O*2 during pregnancy correlates with a 0.9*mm* decrease in birth length [8].

∼

∼

On the other hand, birth outcomes such as birth weight and birth length are strong indicators of fetal and neonatal mortality as well as a variety of other long-term health outcomes. For example, below the ideal birth weight (3, 500 4, 000*g* ), the lower the birth weight, the higher the fetal and neonatal mortality [9, 10].[38](#_bookmark3) In the long run, adults born small or disproportionate (too thin or short) have a high risk for coronary heart disease, high blood pressure, high cholesterol concentrations, and abnormal glucose-insulin metabolism [11]. Children with a birth weight of below 3, 000*g* and a body mass index (BMI) of below 3.4 at birth are associated with reduced visual acuity and impaired hearing [12]. Children with birth weights below 2, 500*g* (conventionally referred to as Low Birth Weight, LBW) have higher rates of subnormal growth and neuro-developmental problems that persist into adolescence [13]. In addition to the health and developmental difficulties faced by individuals, low birth weight can also result in high economic costs for families and society. The expected costs of delivery and initial care for a baby weighing 1, 000*g* at birth can exceed $100, 000 (in 2000 dollars) [14].

∼

The adverse effects of air pollution on birth outcomes may be heterogeneous. For example, [15] find that boys are more affected by ambient air pollution than girls. An average increase of 30*µg* /*m*3 in prenatal exposure to *P M* during pregnancy is associated with a birth weight loss of 189*g* and a birth length loss of 1.1*cm* for male newborns, compared to 17*g* and 0.4*cm* for female newborns. Like other types of pollution, ambient air pollution may be more dangerous for disadvantaged people (in terms of race, income, or education) not only because these people may work and live in areas with high levels of pollution, but also because the adverse effects of air pollution may be nonlinear, and the marginal effects of higher levels of pollution may be greater. Meanwhile, the advantaged may also suffer higher exposure to ambient air pollution due to higher rates of participation in outdoor sports [16].

The epidemiological studies mentioned above use linear or logistic regression models to compare odds ratios between groups with different levels of air pollution exposure, conditional on the demographics of pregnant women. However, family location and time of birth, i.e., the environment to which one is exposed, are chosen by individuals, and the reasons behind these choices are likely to influence birth outcomes as well. Limited by data, this literature often does not control for detailed parental information, making it difficult to determine the reasons for these self-selections. This leads to the possibility of endogeneity problems in these studies, regardless of the precision of the measurement of ambient air pollution concentrations. Thus, the correlations between ambient air pollution and health outcomes identified in the literature are not necessarily causal. In contrast to the literature, I do not exploit the spatial variation in air pollution. Instead, I employ a rich set of spatio-temporal fixed effects to capture all spatial variation in ambient air pollution and compare only infants born at the same location within a specific time interval (e.g., within the same calendar-month).

36A study based on infants born in Oslo, Norway, between 1999 and 2002 found no significant association between full-term birth weight and exposure to traffic pollution (*N O*2 and *P M* ) during pregnancy [2]. I obtained the same results when I restricted the birth outcome data to between 2000 and 2002 (results not shown). This may be due to the small number of observations (26,780 in the literature) and the lack of variation over such a short period of time.

37The study area (Connecticut and Massachusetts, USA) also has very low levels of ambient air pollution. Daily concentrations of *N O*2, *SO*2 and *P M* at the county level are all below 40 *µg* /*m*3 during the study period, meeting most countries’ air quality guidelines.

38Fetal macrosomia (oversized fetus) does not seem to be an issue in my study since it is very rare in the data and has a limited impact on mortality compared to low birth weight.

Furthermore, when it comes to nitrogen oxides (*N Ox* ), the aforementioned papers only examine the effect of ambient *N O*2, whereas little is known about the health effects of ambient *N O* pollution. [39](#_bookmark5) A systematic review of the literature shows that half of the 62 studies on ambient air pollution, birth weight, and preterm birth specify the adverse effects of *N O*2, but no papers examine *N O* specifically [28]. On the website of the American Journal of Epidemiology, one of the top journals in the field of epidemiology, 46 journal articles examine the effects of *N O*2, while only two are related to *N O*, suggesting that research on the adverse effects of ambient *N O* on newborns is inadequate.[40](#_bookmark6) There is a strand of literature that examines *N Ox* as a whole, rather than distinguishing *N O* and *N O*2, because the two pollutants are correlated (often categorized as “traffic pollution") and appear to work together.[41](#_bookmark7) A review of 41 studies on ambient air pollution and birth outcomes mentions three papers that examines *N Ox* , but since ambient *N Ox* mainly includes *N O* and *N O*2 (as well as other nitrogen oxides), it is difficult to know whether *N O* or *N O*2 is important [29].[42](#_bookmark8) To make matters worse, ambient *N O* and *N O*2 concentrations tend to be positively correlated. For example, if only *N O*2 has negative health effects and *N O* does not, then using *N Ox* as the independent variable would underestimate the adverse effects of *N O*2 because part of the variation in *N Ox* is caused by the non-toxic confounder *N O*. In summary, as noted by the World Health Organization (WHO), “Comparisons of *N O* and *N O*2 are scarce and still not conclusive with regard to their relative degree of toxicity” [30]. “Although several studies have attempted to focus on the health risks of *N O*2, the contributing effects of these other highly correlated co-pollutants are often difficult to rule out” [31].

- 1. **Toxicity of** *N O*

Although the effect of ambient *N O* has not been thoroughly examined in the literature, its toxicity makes it dangerous to ignore it as an ambient air pollutant. The toxicology of *N O* is complex. At very low levels, *N O* plays a key role in our cardiovascular, neurological, and immune systems [17]. Low doses of inhaled *N O* therapy is often used as an effective vasodilator in the treatment of certain respiratory diseases.[43](#_bookmark9) This is a reason why ambient *N O* is rarely considered to be hazardous to human health.[44](#_bookmark10)

However, *N O* is far from harmless. Similar to *N O*2, *N O* shows genotoxicity and can induce DNA structural alterations and DNA strand breaks [20].[45](#_bookmark11) What makes *N O* different from other pollutants, such as *N O*2, is its very high affinity for hemoglobin. *N O* has a much greater affinity for hemoglobin than oxygen.[46](#_bookmark12) In blood, *N O* binds to reduced hemoglobin (deoxyhemoglobin) 5 20 times faster than it reacts with oxygen [23]. Therefore, inhaled *N O* that diffuses into our blood through the alveoli and the capillaries will immediately oxidize the Fe(II) of erythrocyte hemoglobin (Hb) to the Fe(III) state, forming methemoglobin (MetHb).[47](#_bookmark13) The increase in methemoglobin impairs oxygen transport due to its lack of ability to bind oxygen reversibly [26]. To make matters worse, the fetus is more likely to be exposed to methemoglobin through the placental barrier [27]. Based on the toxicology of *N O*, it is

∼

39Nitrogen oxides (*N Ox* ) refer to a family of compounds composed of nitrogen and oxygen, such as nitrous oxide *N*2*O*. Regarding ambient air pollution, the two main pollutants in the *N Ox* family are nitrogen dioxide (*N O*2) and nitric oxide (*N O*) because of their high toxicity to human health. In gaseous form, *N O*2 has a reddish-brown color and a strong odor and is a major component of visible photo-chemical smog. In contrast, *N O* is a colorless gas with a sweet odor.

40These two studies find that the increase in ambient *N O* exposure during pregnancy is associated with a higher risk of low birth weight [32] and childhood acute lymphoblastic leukemia [33]. If we expand the topic from “birth outcomes" to general health outcomes, there are studies on the relationship between *N O* and diseases such as asthma [34].

41Despite having the same origin and being interrelated, *N O* and *N O*2 have very different toxicity to human health and

different temporal variations. The toxicity, chemical properties and temporal variation of these two pollutants are discussed in sections [A.2](#_bookmark4) and [A.3](#_bookmark14).

42It should be noted that the notation of *N O* ambiguously represents *N Ox* in this literature review, whereas the notation *N Ox*

is used in the other papers and in my study.

43The safe dose of inhaled *N O* (i*N O*) therapy in neonates has not been fully established, but most studies start with a dose of 25 *µg* /*m*3 and gradually decrease the dose. A does of 50 *µg* /*m*3 has been used in adults. See also: [18] and [19]. In my study, the ambient *N O* concentration can be much higher than such a level.

44Another reason is that *N O* is relatively unstable and can be oxidized to *N O*2 by *O*2 and *O*3 in the ground atmosphere. This

will be discussed in Subsection [A.3](#_bookmark14).

45 *N O*2 is also known to induce DNA mutations and strand breaks in the respiratory tract [21]. Except for genotoxicity, the effects of *N O*2 seem to be limited to the respiratory system. Inhalation of high concentration of *N O*2 may result in acute bronchospasm, delayed pulmonary edema, and late bronchiolitis obliterans. Chronic exposure to low concentrations of *N O*2 appears to induce pulmonary fibros and inhibit pulmonary defense mechanisms [22].

46 *N O* has a 1500 times higher affinity for hemoglobin than *CO*, another air pollutant that is known to have a high hemoglobin affinity and impede the transport of oxygen [23].

47The increase in maternal methemoglobin is also a biomarker for determining when a pregnant woman’s health is threatened by toxic substances in the environment [24]. In fact, methemoglobinemia is a well-known side effect of nitric oxide therapy mentioned above, and this therapy requires close monitoring for methemoglobin level [25].

important to investigate whether prenatal exposure to ambient *N O* may adversely affect the health status of the newborn. In a broader sense, my research also adds new evidence to the literature on ambient air pollution and human health.

In addition, recent studies are beginning to realize the role of genetic pleiotropy in birth outcomes. That is, birth weight loss and other long-term health problems may be the results of certain genetic defects. For example, children of diabetic fathers are, on average, lighter than children of non-diabetic fathers [44, 45], and infants of mothers at risk for late onset diabetes are heavier [46]. The genotoxicity of *N O* and *N O*2 and the evidence of a positive association between air pollution and the risk of type II diabetes [47, 48, 49] make it worthwhile to investigate whether genetic pleiotropy is a mechanism by which ambient air pollution reduces birth weight. The established literature on air pollution and neonatal health outcomes is understudied on this issue. At the same time, omitting heritable traits would lead to omitted-variable bias when ambient air pollution exposure is associated with certain parental genetic characteristics. In this paper, with the rich registry data, I can observe the parents’ history of diabetes, which helps me overcome this problem of omitted variable bias.

- 1. **Ambient** *N O* **and** *N O*2 **concentration**

There are two main obstacles to examining the effect of ambient *N O* in the literature: (i) The concentrations of *N O* and *N O*2 are highly correlated. (ii) *N O* is less toxic at very low concentrations compared with *N O*2, and in many areas studied in the literature, ambient *N O* concentrations are lower than *N O*2. Interestingly, the characteristics of the Norwegian ambient air pollution depicted in Figure [A5](#_bookmark20) make it possible to overcome these two obstacles.[48](#_bookmark15)

The gray and brown curves in Figure [A5](#_bookmark20). Part A Figure of [A5](#_bookmark20) shows the temporal variation of monthly average ambient *N O* and *N O*2 concentrations in Norway from 1999 to 2016. It is clear that *N O* and *N O*2 concentrations are strongly seasonal and often positively correlated.[49](#_bookmark16) However, the amplitude of the *N O* concentration curve is larger than the variation of *N O*2, i.e., the fluctuation of *N O* concentration is larger than that of *N O*2 during one year. Specifically, in winter, both *N O*2 and *N O* concentrations showed an increasing trend, but the increase in *N O* is greater; in summer, both *N O*2 and *N O* concentrations showed a decreasing trend, but the decrease in *N O* is again greater. The different seasonal variations in *N O* and *N O*2 levels imply that infants born in the same calendar quarter or even calendar-month may be exposed to ambient air with different *N O*/*N O*2 ratios in the last trimester of pregnancy, a critical period for the fetus. This difference allows me to study the effects of these two pollutants separately.

The seasonal fluctuations of *N O* and *N O*2 are mainly caused by photochemical reactions and ambient ozone (*O*3). In the terrestrial atmosphere, *N O* is less stable than *N O*2 and can be rapidly oxidized to *N O*2 by *O*3.[50](#_bookmark17) Therefore, *N O* is known as a precursor of *N O*2. Since *O*3 at the ground level is formed mainly through photochemical reactions, when summer temperatures and solar irradiance are high, active photochemical reactions increase the concentration of *O*3 in the environment, resulting in more *N O* being oxidized to *N O*2 [36].[51](#_bookmark18) In winter, low temperatures and low solar irradiance prolong the life time of both *N O* and *N O*2 in the atmosphere.[52](#_bookmark19) Less vegetation activity and higher use of heating energy in winter also contribute to high ambient *N O* and *N O*2 concentrations.

48It should be noted that the detection of *N O* and *N O*2 is not difficult. Sensors for accurate detection of *N O* using chemilu- minescent metho have been commercially available since the 1970s. Taking the example of air pollution in California, which has been studied extensively in the literature. From the database provided by [California Environmental Protection Agency](https://calepa.ca.gov/), we can easily obtain hourly average concentrations of ambient *N O*, *N O*2 and other *N Ox* for counties such as Los Angeles and San Diego as early as 1963.

49Since ambient *N O* and *N O*2 are products of the reaction of nitrogen and oxygen at high temperatures, such as combustion

processes in motor vehicles, power plants, and manufacturing, these two ambient air pollutants are positively correlated with each other. In Norway, domestic shipping accounts for about one-third of *N Ox* emissions. Oil/gas extraction and road traffic each account for a quarter of total *N Ox* emissions. The rest of the *N Ox* emissions are contributed by industrial (10%) and agricultural (3%) production, among others. For more information, please see: <https://www.ssb.no/statbank/table/08941/>.

50Ground level ambient *O*3 is another environmental pollutant that can damage the human respiratory system due to its

strong oxidizing power. Although both oxygen (*O*2) and *O*3 can oxidize *N O* to *N O*2, *O*2 and *N O* react very slowly in air. In the laboratory, *O*2 oxidizes slowly (in days) to *N O*2 at room temperature when *N O* is at a concentration of 100*µg* /*m*3, while *O*3 can complete the oxidation process in a few hours [35].

51Precisely, in the ground atmosphere, with the participation of sunlight irradiation and other pollutants, relatively small amounts of *N O*2 can in turn be decomposed into atomic oxygen (rapidly forming *O*3) and *N O*, due to photolysis. The level of *N Ox* concentration in the environment detected by the air quality sensor is actually in dynamic equilibrium (the so-called photostasis).

52In tunnels with low solar irradiation and O 3 levels, the ambient *N O* concentration is often 5-10 times higher due to weak photochemical reactions. This has been documented by the Norwegian Public Roads Administration: <https://vegvesen.brage.unit.no/vegvesen-xmlui/handle/11250/2656305>.


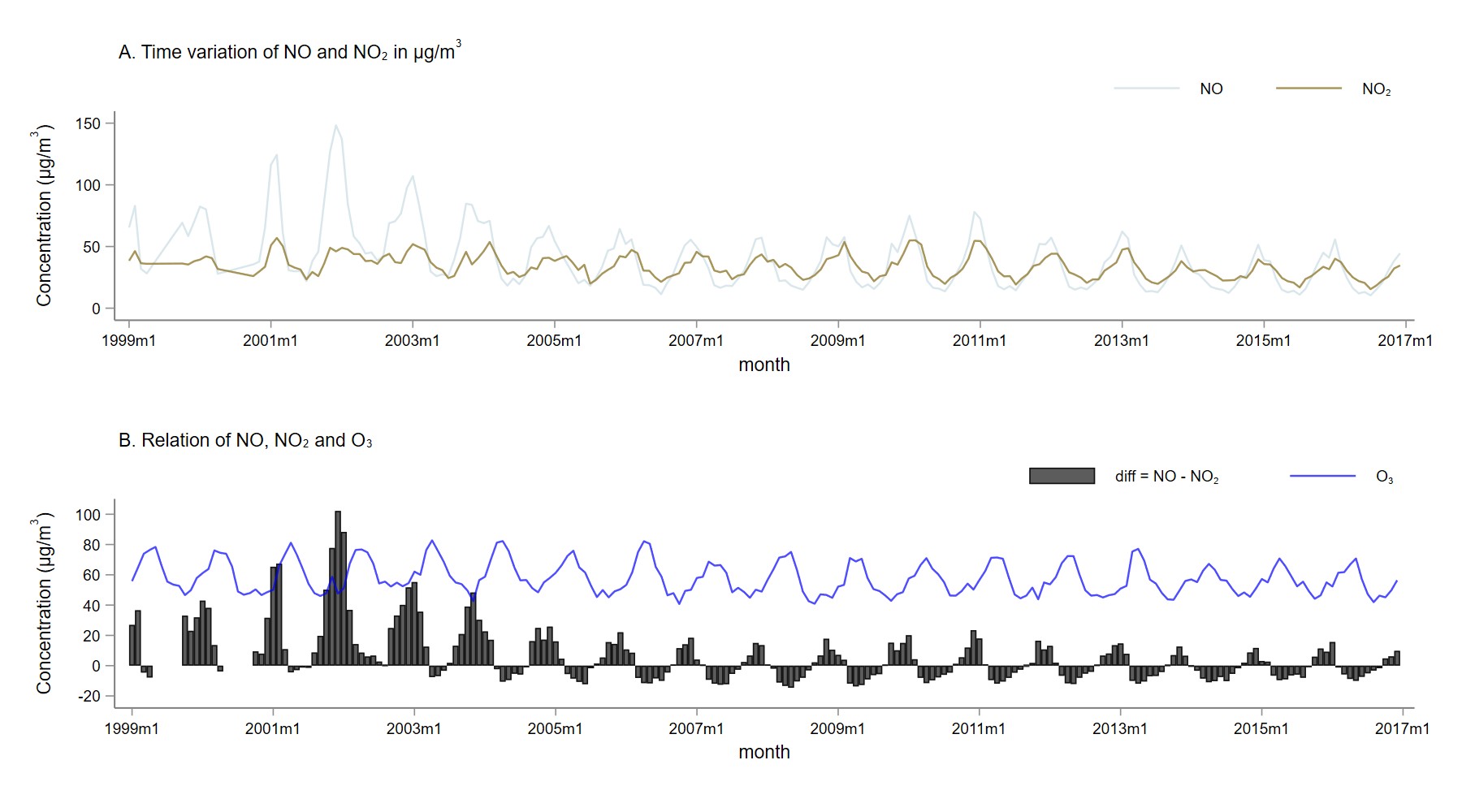


Figure A5: Monthly average *N O*, *N O*2 and *O*3 concentrations in Norway, 1999-2016

The above relationships between *N O*, *N O*2 and *O*3 are shown in Panel B of Figure [A5](#_bookmark20). The black bars in Panel B indicate the difference between *N O* and *N O*2 (i.e., *N O N O*2 in *µg* /*m*3), while the blue curve indicates the ambient *O*3 concentration. This may be more pronounced at high latitudes (e.g., Norway) because photochemical reactions are much weaker in cold and dark winters [37], while long solar days promote photochemical reactions in summer [38].

−

It is noteworthy that the concentration of *N O* has dropped sharply since 2005, as shown in Figure [A5](#_bookmark20). This appears to be the result of new air pollution regulations in Norway, which came into effect in 2002, replacing regulations established in 1997. In addition to establishing stricter ambient air pollution limits, the new regulations control previously unregulated pollutants. Under the new regulations, certain air quality objectives must be met between 2005 and 2010. [53](#_bookmark21)

Another feature that distinguishes Norway from other regions is that the annual average ambient *N O* concentrations are higher than *N O*2. In more than 30 European cities, ambient *N O* concentrations are lower than *N O*2 [39, 40].[54](#_bookmark22) As mentioned above, in New Jersey and Las Vegas, the annual average concentration of ambient *N O* is only about 1/4 of *N O*2 [41, 42]. Given that *N O*2 is more toxic than *N O* at low concentrations, ambient *N O* pollution is less important in these areas. Nevertheless, in Figure [A5](#_bookmark20), we can find that in most cases, the ambient *N O* concentrations in Norway are similar to or even higher than *N O*2. Until 2005, ambient *N O* concentrations are higher than *N O*2 almost all year round. In 2002, *N O* concentrations are even three times higher than *N O*2. This suggests that the environmental *N O* problem may be more severe in Norway than in other countries.

In addition to the weak photochemical reactions, the unusually high *N O*/*N O*2 ratio in Norway can be explained by the chemical properties of *N O* and *N O*2 and the special climate of Norway. The freezing and condensation points of *N O*2 are about 21°*C* and 10°*C*, respectively. In Norway, the average monthly temperature is below 10°*C* for 8 months of the year. Even in summer, the maximum monthly average temperature stays below 21°*C*. Although *N O*2 exists in gaseous form in air under normal ambient conditions due to its low partial pressure in the atmosphere (908*mmH g* at 25°*C* ) [43], the gas will be compressed and much heavier than air if enough *N O*2 molecules are present in the ambient air. Therefore, *N O*2 is more common in low-lying areas. In contrast, *N O* is lighter than *N O*2,

−

and its condensation point of −152°*C* means that its density is less affected by the ambient climate at the surface.

53More information about the new regulations (in Norwegian) can be found at: [https://lovdata.no/dokument/LTI/forskrift/20](https://lovdata.no/dokument/LTI/forskrift/2002-10-04-1088)02- [10-04-1088](https://lovdata.no/dokument/LTI/forskrift/2002-10-04-1088).

54The two exceptions in the literature are Athens and Glasgow, which have a *N O*/*N O*2 ratio of 1.22 ∼ 1.39, but both cities have very high air pollution levels (*N Ox* > 150*µg* /*m*3 per day), unlike Norway.

In conclusion, the above discussion implies that ambient *N O* may be a serious ambient air pollutant in Norway (and possibly in other high-latitude areas). The high concentration of ambient *N O* relative to *N O*2 and the different seasonal patterns of variation of the two pollutants make it possible to determine their health effects separately.

# **Performance of IDW interpolation**

The IDW interpolation method weights the monitoring values within a certain range (radius) of the given points (centroids of *g r unnkr e t s*). If the radius is short, IDW interpolation relies only on detection stations close to *grunnkrets*, so the interpolated values are closer to the true values; a long radius covering more stations may involve more measurement errors because some stations are too far from the *grunnkrets* to precisely interpolate. On the other hand, the radius also determines how many *grunnkrets* (and thus observations) I can interpolate the air pollution concentration to, since there may not be any detection stations within a short range of a *grunnkrets*. Estimates of the effect of air pollution on birth outcomes may be less precise in this case due to the lack of observations. In other words, the choice of radius thus involves a trade-off between interpolation (measurement) accuracy and identification accuracy (sample size).

I use a cross-validation strategy to test the performance of the IDW method at different radii and distance weights (exponent *e* in Function 1). For a monitoring station *i* that detects a pollution value *pr e al* in week *t*, I first interpolate the pollution value *pi nt er p* at the same site where *i* is located using the IDW method. The interpolation is based on other stations within a certain radius (except for station *i* itself ). I then compare the interpolated value *pi nt er p* with the true value *pr e al* detected at station *i*. Intuitively, the correlation between the interpolated values and the detected true values shows how well the interpolation performs. Again, since I will use *grunnkrets*-(calendar) month fixed effects in my identification strategy, cross-validation should take these fixed effects into account. Formally, I

*i t*

*i t*

*i t*

*i t*

regress the actual value *pr e al* detected by the station on the interpolated value *pi nt er p* (both at the weekly level),

*i t i t*

conditional on the station’s location and the (calendar) month fixed effects (and the interaction of the two), as shown in Eq. [1](#_bookmark23).

*pr e al* = *pi nt er p* · *b* + *si* + *mt* + *si* · *mt* + *ϵi t* (1)

*i t*

*i t*

Where *b* is the regression coefficient of the interpolated value *pi nt er p* . *si* and *mt* are the fixed effects of monitoring stations and (calendar) months, and *si mt* is the interaction effect. Both real and interpolated pollutant concentra- tion data are at the calendar-week level (approximately 700 unique calendar-weeks). The *R*2 value of the regression [1](#_bookmark23) is the square of the correlation coefficient, which describes how well the interpolated and fixed effects work.

·

Table[B1](#_bookmark25) shows the regression *R*2 values for different distance weights (index *e* in Function 1) and radii for some major ambient air pollutants.[55](#_bookmark24) With the exception of *P M*2.5 in column (6), the IDW interpolation together with fixed effects predicts well the actual pollutant concentrations (*R*2 ≈ 90%).

The power of the distance in column (1), *e*, is used as a penalty for the distance between the site and the location to be interpolated. The intuition is that although adding more sites provides more information, the interpolation may be distorted by sites that are too far away. According to Table [B1](#_bookmark25), larger distance indices *e* tends to slightly reduce the fit, suggesting that I should not penalize the addition of monitoring stations too much. In other words, adding more monitoring stations is relatively beneficial to improving the fit.

The radius in column (2) has little effect on the fit because most of the monitoring stations are located near large cities that are much farther apart than the radius itself. Therefore, increasing the radius from 10 miles to 40 miles may not include more monitoring stations. In addition, pollutant concentrations within a city can be highly correlated. Adding more stations within a city would not greatly improve interpolation.

Note that both the concentrations of air pollutants and spatial fixed effects are at the monitoring station level, so all spatial variation in pollutant concentrations is captured by fixed effects. The *R*2 actually describes how well the interpolation predicts the temporal variation (within a calendar-month) of the actual pollutant concentration, or in other words, *R*2 is the correlation index between the interpolated values and the actual concentrations after the fixed effects are excluded. My identification in the next section exploits the temporal variation in prenatal exposure to ambient air pollution in exactly the same way.

I also cross-validated the performance of the IDW method for interpolation of meteorological conditions. Because there are more meteorological monitoring stations in Norway, the interpolation is also more accurate (*R*2 85%), as shown in Table [B2](#_bookmark28). Based on the cross-validation results in Table [B1](#_bookmark25) and Table [B2](#_bookmark28), I used 20 miles (32*km*) as the

≥

55Because the shortest distance between stations monitoring *O*3 is greater than 10 miles, the cross-validation of *O*3 is not

applicable for a radius of 10 miles.

Table B1: Cross-validation *R*2: how well the interpola- tion and fixed effects predict the real concentration (%)

| *e* | radius | *N O* | *N O*2 | *P M*10 | *P M*2.5 | *O*3 |
| --- | --- | --- | --- | --- | --- | --- |
|  | 10 | 86.9 | 89.3 | 79.8 | 38.0 | - |
|  | 15 | 87.2 | 90.1 | 80.1 | 38.3 | 91.9 |
| 0.1 | 20 | 87.2 | 90.1 | 80.1 | 38.4 | 89.3 |
|  | 25 | 87.2 | 90.1 | 80.3 | 38.4 | 89.3 |
|  | 30 | 87.2 | 90.1 | 80.5 | 38.5 | 89.3 |
|  | 10 | 86.7 | 89.1 | 79.8 | 38.0 | - |
|  | 15 | 87.1 | 89.9 | 79.8 | 38.3 | 91.9 |
| 1 | 20 | 87.1 | 90.0 | 79.9 | 38.3 | 89.3 |
|  | 25 | 87.0 | 90.0 | 80.0 | 38.3 | 89.3 |
|  | 30 | 87.0 | 90.0 | 80.1 | 38.4 | 89.3 |
|  | 10 | 86.3 | 88.7 | 79.5 | 38.0 | - |
|  | 15 | 86.7 | 89.6 | 79.5 | 38.2 | 91.9 |
| 2 | 20 | 86.7 | 89.6 | 79.6 | 38.2 | 89.3 |
|  | 25 | 86.7 | 89.7 | 79.7 | 38.2 | 89.3 |
|  | 30 | 86.6 | 89.7 | 79.8 | 38.3 | 89.3 |
|  | 10 | 85.3 | 88.0 | 78.9 | 37.8 | - |
|  | 15 | 85.7 | 89.0 | 79.0 | 38.0 | 91.9 |
| 5 | 20 | 85.7 | 89.0 | 79.0 | 38.0 | 89.3 |
|  | 25 | 85.7 | 89.1 | 79.1 | 38.1 | 89.3 |
|  | 30 | 85.7 | 89.1 | 79.3 | 38.1 | 89.3 |

Notes: (1) As defined in Section 3.1, *e* is the power of distance as defined in equation; radius determines within which range the monitoring stations are used for interpolation. (2) Column 3-7 contains the *R*2 of regression [1](#_bookmark23) for different pollutants, which shows how well the real concentration is explained by the interpolation and fixed effects (3) There is no interpolation for *O*3 with a radius of 10 miles because there is no station within such a range because *O*3 is only monitored by a few stations. For the same reason, I didn’t cross-validate *SO*2 either.

base radius (which is also the same as [52]) and 0.1 as the default distance power, which means that the weighting is fairly uniform (i.e., close to the arithmetic mean).[56](#_bookmark26)

However, the interpolation method always leads to some measurement error. Even in the cross-validation in Table [B1](#_bookmark25), the interpolation and fixed effects do not perfectly (*R*2 100%) predict the actual concentration.[57](#_bookmark27) If the measurement error are random (classical), it would bias my estimates towards zero. However, the measurement error may not be random. Because air pollution monitoring stations are mainly located near major roads in large cities, where ambient air pollutant concentrations may be higher and more volatile, interpolation may overestimate concentration levels and volatility in areas relatively far from the monitoring stations. This is particularly true for *N O*, which is more likely to be oxidized in the ambient air after emission, as described in Section [A](#_bookmark0). An overestimate of *N O* also biases the estimate toward zero (or even have a protective effect) if people living relatively far from the main road are wealthier and have better (potential) birth outcomes.

=

56In Appendix Section [C](#_bookmark29) I also tried different radii and distance weighting indices *e* to check the robustness of my identification strategy.

57When it comes to identification in Section 4, since I also conditioned on the (interpolated) average meteorological conditions, which are related to the real pollutant concentrations, part of the measurement error can be taken into account.

Table B2: Cross-validation *R*2: how well the interpolation and fixed effects predict the real weather condition (%)

| p | miles | *humi* | *pr eci p* | *pr e ss* | *t emp* | *w i nd* |
| --- | --- | --- | --- | --- | --- | --- |
|  | 10 | 91.4 | 88.7 | 100.0 | 99.3 | 89.2 |
|  | 15 | 90.9 | 85.8 | 100.0 | 99.2 | 88.6 |
| 0.1 | 20 | 90.5 | 86.1 | 100.0 | 99.2 | 88.8 |
|  | 25 | 88.6 | 85.1 | 99.9 | 99.1 | 89.1 |
|  | 30 | 87.6 | 80.4 | 99.9 | 99.1 | 90.9 |
|  | 10 | 91.5 | 88.7 | 100.0 | 99.2 | 89.2 |
|  | 15 | 90.8 | 85.8 | 100.0 | 99.2 | 88.6 |
| 1 | 20 | 90.4 | 86.2 | 100.0 | 99.1 | 88.7 |
|  | 25 | 88.7 | 85.3 | 99.9 | 99.1 | 89.0 |
|  | 30 | 87.7 | 80.6 | 99.9 | 99.1 | 90.8 |
|  | 10 | 91.5 | 88.5 | 100.0 | 99.2 | 89.2 |
|  | 15 | 90.8 | 85.7 | 100.0 | 99.2 | 88.6 |
| 2 | 20 | 90.3 | 86.1 | 100.0 | 99.1 | 88.6 |
|  | 25 | 88.6 | 85.2 | 99.9 | 99.1 | 88.9 |
|  | 30 | 87.6 | 80.6 | 99.9 | 99.1 | 90.6 |
|  | 10 | 91.5 | 88.2 | 100.0 | 99.2 | 89.2 |
|  | 15 | 90.7 | 85.4 | 100.0 | 99.1 | 88.5 |
| 5 | 20 | 90.2 | 85.6 | 100.0 | 99.1 | 88.4 |
|  | 25 | 88.4 | 84.6 | 99.9 | 99.0 | 88.6 |
|  | 30 | 87.3 | 80.1 | 99.9 | 99.0 | 90.3 |

Notes: (1) As defined in Section 3.1, *e* is the power of distance as defined in equation; radius determines within which range the mon- itoring stations are used for interpolation. (2) Column 3-7 contains the *R*2 of in regression [1](#_bookmark23) for different meteorological conditions, which shows how well the real conditions is explained by the inter- polation and fixed effects.

# **Robustness check**

In this section, I first evaluate the sensitivity of my identification strategy to IDW interpolation, which affects both estimation and statistical inference (as it affects sample size). I then indirectly test the conditional independence assumptions underlying my identification strategy by testing for spatio-temporal fixed effects and other potential confounders. Finally, I discuss the case of mothers moving pre/post-natally, which may lead to measurement error and make spatial fixed effects a “bad control” [59].

- 1. **Sensitivity to IDW interpolation**

The choice of the radius for the IDW interpolation method is a trade-off between the accuracy of the interpolation itself and the precision of the estimate (because it affects sample size), as discussed in Section 3. The shorter the distance between the location to be interpolated and the monitoring station, the closer the interpolated value will be to the actual value detected by the station; on the other hand, the shorter the distance, the fewer the monitoring stations around the location, and therefore the fewer the locations (observations) that can be covered by the interpolation.

Table C1: Benchmark regression with different IDW radius

|  | (1) | (2) | (3) | (4) | (5) | (6) | (7) |
| --- | --- | --- | --- | --- | --- | --- | --- |
| radius: | 10 | 15 | baseline | 25 | 30 | 35 | 40 |
| A. Birth  *N O* | weight  -1.263∗ | -1.348∗∗ | -1.387∗∗ | -1.358∗∗ | -1.036∗ | -0.891 | -0.687 |
|  | (0.691) | (0.647) | (0.611) | (0.595) | (0.569) | (0.558) | (0.550) |
| *N O*2 | 1.914 | 0.136 | -0.259 | -0.288 | -1.233 | -1.187 | -1.225 |
|  | (2.002) | (1.889) | (1.762) | (1.692) | (1.591) | (1.530) | (1.515) |
| *P M*10 | 1.460 | 1.486 | 1.329 | 2.660∗ | 2.284 | 2.389∗ | 2.627∗ |
|  | (1.721) | (1.581) | (1.489) | (1.452) | (1.413) | (1.383) | (1.361) |
| *r* 2 0.448 | | 0.453 | 0.464 | 0.469 | 0.474 | 0.479 | 0.483 |
| Obs. | 167,713 | 200,554 | 225,239 | 244,918 | 261,907 | 275,778 | 290,303 |
| B. Birth length | | | | | | | |
| *N O* | -0.031 | -0.047 | -0.052∗ | -0.063∗∗ | -0.044 | -0.035 | -0.029 |
|  | (0.034) | (0.031) | (0.029) | (0.028) | (0.027) | (0.026) | (0.026) |
| *N O*2 | 0.031 | -0.031 | -0.023 | -0.020 | -0.054 | -0.050 | -0.045 |
|  | (0.092) | (0.086) | (0.080) | (0.076) | (0.072) | (0.069) | (0.068) |
| *P M*10 | -0.006 | 0.007 | -0.004 | 0.023 | 0.012 | 0.015 | 0.023 |
|  | (0.090) | (0.083) | (0.077) | (0.074) | (0.072) | (0.070) | (0.069) |
| *r* 2 0.446 | | 0.450 | 0.461 | 0.468 | 0.472 | 0.476 | 0.480 |
| Obs. | 159,599 | 190,484 | 212,938 | 231,167 | 246,758 | 259,498 | 273,088 |

Notes: (1) IDW radius is defined in Section 3 radius in miles. (2) All regressions are based on the benchmark model (column (7) of Table 3 and Table 4). (3) Cluster robust standard errors at *grunnkrets* level in parentheses. (4) *** *p* 0.01, ** *p* 0.05, * *p* 0.1. (5) Pollutants in *µg* /*m*3, birth-weight in gram, birth length in millimeter.

< < <

In Table [C1](#_bookmark30), I try radii from 10 to 40 miles to check how sensitive my estimates are to the choice of radius. Panels A and B of Table [C1](#_bookmark30) have birth weight and birth length as explanatory variables, respectively. The regression results in column (4) of Table [C1](#_bookmark30) are the same as the baseline regression results (column (7) of Table 3 and Table 4), i.e., both have a radius of 20 miles. For simplicity, I report only the coefficients for the three pollutants in Table [C1](#_bookmark30). We can see that the effect of *N O* on birth weight is fairly stable and remains significant at the 5% level when the radius is between 15 and 25 miles (columns (2)-(5)). The coefficient of the effect of *N O* on birth length is about 0.05*mm* when the radius is between 15 and 25 miles, but is significant at the 5% level only when the radius is 25 miles. The significant effect of prenatal *N O* exposure disappears in both Panel A and Panel B when the radius exceeds 30 miles. This is because interpolation tends to overestimate the true pollutant fluctuations as the radius increases, as discussed in Section 3. In summary, identification is not sensitive to interpolation when the radius is

−

between 15 and 25 miles.

- 1. **Validity of conditional independence**

My identification relies on controlling for a rich set of spatio-temporal fixed effects. As described in chapter 4, to claim that the coefficients on pollutants are their causal effects on birth outcomes, prenatal air pollution exposure in the last trimester of pregnancy should be randomly assigned conditional on fixed effects and other covariates, i.e., conditionally independent. The assumption of conditional independence is more convincing when the resolution of spatial and temporal fixed effects is higher, because in this case there is less room for household self-selection of place of residence and time of delivery. On the other hand, however, fewer babies are born in the areas and time intervals determined by the high-resolution fixed effects. Therefore, the estimates are less precise. The choice of the resolution of the spatio-temporal fixed effects involves a trade-off between the bias and precision of the estimates.

Table C2: Regression of *N O* concentration in the 3rd. trimester on parental characteris- tics and F-test

| (1) | | (2) | (3) | (4) | (5) | (6) |
| --- | --- | --- | --- | --- | --- | --- |
| no FE | | TWFE | *p*-*q* | *p*-*m* | *g* -*q* | baseline |
| A. F-test: *N O* as dependent variable | | | | | | |
| *r* 2 0.581 | | 0.848 | 0.948 | 0.986 | 0.960 | 0.991 |
| Obs. 371,646 | | 371,531 | 365,104 | 326,562 | 320,343 | 226,455 |
| *F* (14, *d fr* ) 115.95∗∗∗ | | 2.78∗∗∗ | 2.52∗∗∗ | 1.33 | 1.70∗∗ | 1.46 |
| *d fr* 4,264 | | 4,149 | 1,185 | 1,161 | 3,784 | 3,527 |
| B. Birth weight  *N O* 0.573∗∗∗ | | -0.437∗∗∗ | -0.839∗∗∗ | -0.927∗∗ | -0.786∗∗∗ | -1.387∗∗ |
| (0.085) | | (0.141) | (0.210) | (0.452) | (0.247) | (0.611) |
| *N O*2 -1.616∗∗∗ | | -0.133 | 0.101 | -0.270 | -0.118 | -0.259 |
| (0.186) | | (0.280) | (0.489) | (1.185) | (0.577) | (1.762) |
| *P M*10 1.975∗∗∗ | | 0.462 | 1.752∗∗∗ | 2.137∗ | 1.308∗∗ | 1.329 |
| (0.225) | | (0.311) | (0.521) | (1.132) | (0.632) | (1.489) |
| *r* 2 0.015 | | 0.034 | 0.172 | 0.324 | 0.315 | 0.464 |
| Obs. | 369,208 | 369,092 | 362,715 | 324,271 | 318,365 | 225,239 |
| C. Birth length | | | | | | |
| *N O* | 0.030∗∗∗ | -0.003 | -0.024∗∗ | -0.023 | -0.029∗∗ | -0.052∗ |
|  | (0.004) | (0.006) | (0.010) | (0.021) | (0.012) | (0.029) |
| *N O*2 | -0.000 | -0.013 | -0.016 | -0.094∗ | -0.003 | -0.023 |
|  | (0.009) | (0.012) | (0.022) | (0.053) | (0.026) | (0.080) |
| *P M*10 | 0.000 | -0.014 | 0.040 | 0.065 | 0.030 | -0.004 |
|  | (0.010) | (0.014) | (0.025) | (0.054) | (0.028) | (0.077) |
| *r* 2 0.009 | | 0.032 | 0.174 | 0.324 | 0.315 | 0.461 |
| Obs. | 355,425 | 355,311 | 348,653 | 309,678 | 304,128 | 212,938 |

Notes: (1) Panel A is derived from regressions of average ambient *N O* concentration in the third trimester on parental characteristics and weather conditions with different sets of spacial- temporal fixed effects: “no FE” conditioned on no fixed effects; “TWFE” means calendar month and *grunnkrets* are controlled for. In column 3-6, *q* and *m* represent calendar quarter and calendar month fixed effects; *g* and *p* mean *grunnkrets* and post-zone fixed effects (both main effect and the interaction are controlled for). The F statistic is to test the joint significance of the 14 parental characteristic variables, where *d fr* is the residual degrees of freedom. The independent variables in Panel B and Panel C are birth weight and birth length separately. (2) income and wealth in million Norwegian kroner at current price. (3) Cluster robust standard errors at *grunnkrets* level in parentheses in column 1,2,5 and 6; the standard errors are cluster at post-zone level in column 3-4. (4) *** *p* < 0.01, ** *p* < 0.05, * *p* < 0.1.

Although I cannot test the conditional independence hypothesis directly, I can infer its validity indirectly from the characteristics of the parents. In Table [C2](#_bookmark31), Panel A, I regress mean ambient *N O* concentrations in the last trimester on the 14 observable parental characteristics and weather conditions used in the baseline regression. Also, I control for spatio-temporal fixed effects at different resolutions in these regressions. The spatio-temporal fixed effects used in the first two columns of Table [C2](#_bookmark31) are: (1) “no FE” for no fixed effects; (2) “TWFE” for calendar-month and *grunnkrets* fixed effects (without interaction term); In columns (3)-(6) of Table [C2](#_bookmark31) , the main effect as well as the interaction of post-zone *p* (or *grunnkrets g* ) and calendar quarter *q* (or calendar-month *m*) are controlled

for. If prenatal exposure to ambient air pollution is randomly assigned conditional on these fixed effects and weather conditions, then the parental characteristics should not be jointly significant. Therefore, I tested the joint significance of the 14 parental characteristics after regression.

According to Table [C2](#_bookmark31), once the fixed effects of postcode-(calendar)month or *grunnkrets*-(calendar)month are controlled for (columns (4) and (6)), the *F* -statistic for Panel A becomes insignificant at the 10% level. This means that we cannot reject the hypothesis that the 14 parental characteristics are jointly independent of prenatal *N O* exposure at that level of significance. In other words, in terms of parental characteristics, prenatal exposure to *N O* appears to be randomly assigned to infants when postcode-(calendar) months or *grunnkrets*-(calendar) months are given, rather than chosen by the parents themselves. In contrast, in Table [C2](#_bookmark31), columns (1)-(3) and (5), the *F* statistic is significant at the 0.1% and 5% levels, implying that prenatal *N O* exposure is associated with the characteristics of the infant’s parents under the condition of lower resolution of fixed effects, when the conditional independence assumption does not seem to hold.[58](#_bookmark32)

While the zip-code-(calendar) month and *grunnkrets*-(calendar) month fixed effects perform fairly well in Table [C2](#_bookmark31) Panel A, the former regression has more observations than the latter. This is because in a given calendar-month, a zip-code area is more likely to have enough observations for estimation, whereas in the smaller *grunnkrets*, there may be too few births to estimate (i.e., more singletons). Such singletons that do not participate in the regression are more likely to occur due to the low population density in rural areas. Thus, when controlling for *grunnkrets*- (calendar)month fixed effects, the observations represent more urban areas. In contrast, when controlling for postcode-(calendar)month fixed effects, more rural samples are able to participate in the regression due to fewer singletons. In my study, unless otherwise stated, I reserve the use of *grunnkrets*-(calendar) month fixed effects in order to avoid as much as possible the selection of unobservables, especially in an area with many newborns in the zip-code area.

In panels B and C of Table [C2](#_bookmark31), I regress birth outcomes on the covariates in the baseline regression using different levels of fixed effects. By comparing columns (4) and (6) with the other columns, we can see that the effect of *N O* is greatly underestimated when the resolution of the fixed effects is relatively low. This happens for a number of reasons, including the choice of living location and delivery date, or the random measurement error of the IDW interpolation is accentuated at coarser fixed effects, as explained in Section 3.[59](#_bookmark33) It is worth noting that the effect of environmental *N O* is stronger and more pronounced in column (6) of Table [C2](#_bookmark31) than in column (4), which may be due to the random measurement error described above, but there is another possible explanation: the effect of ambient air pollution may be heterogeneous when the samples are different. As mentioned earlier, the sample in participating in the regression in column (6) is more representative of infants born in urban areas with higher levels of pollution. The marginal effect of air pollution on this group of infants may be larger.[60](#_bookmark34)

- 1. **Potential confounders**

Ambient air pollutants are usually produced simultaneously by traffic and industrial production. As a result, the concentrations of these pollutants are closely correlated. In addition, the complex interactions between ambient air pollutants make it challenging to disentangle their respective effects on birth outcomes. Therefore, I further introduced three other pollutants, *P M*2.5, *O*3, and *SO*2, in my regressions to check whether my baseline regressions are influenced by these omitted variables. As suggested by Subsection [A.3](#_bookmark14), it is of particular interest to examine how the correlation between *O*3, *N O* and *N O*2 affects the birth results. In addition, in Table [C3](#_bookmark35), I also tried to include only these three pollutants mentioned above in the baseline regression model, and I find that only *N O* have a significant effect on birth weight and birth length.

In Table [C4](#_bookmark36), panels A and B examine the effects of environmental pollutants on birth weight and birth length, respectively. For comparison purposes, I replicate the baseline regression results (column (7) of Table 3 and Table 4) in the first column of Table [C4](#_bookmark36). I include the average concentrations of *P M*2.5, *O*3, and *SO*2 for the third trimester in columns (2) to (4) of Table [C4](#_bookmark36) in the baseline regression, respectively. In column (5), I control for *O*3 and

×

58The extraordinarily high *R*2 values when the interaction term *m g* and weather conditions are controlled for in column (6) are unsurprising given that with the *grunnkrets*-(calendar) month fixed effects conditioned on, the only variation left in prenatal *N O* exposure originates from the variation of birth date within a month. I thus need a large sample to provide enough power for a precise estimation.

59Similar to the measurement bias of the Section 3 difference increasing with radius, in the case of more rural samples participating in the regression, I may have underestimated the *N O* coefficient by overestimating the pollution fluctuations in rural areas.

60In Section 6, I find that a one-unit increase in ambient *N O* has a greater adverse effect on birth outcomes when pollution levels are higher.

Table C3: The effects of ambient *O*3 in the 3rd. trimester on birth outcomes

| (1) | | (2) | (3) | (4) |
| --- | --- | --- | --- | --- |
| A. Birth weight | |  |  |  |
| *O*3 | 0.911 | -0.141 | -0.121 | -0.335 |
|  | (0.955) | (1.143) | (1.370) | (1.383) |
| *N O* |  | -0.985∗ | -1.006 | -1.796∗ |
|  |  | (0.529) | (0.769) | (1.038) |
| *N O*2 |  |  |  | 2.808 |
|  |  |  |  | (2.528) |
| *r* 2 0.439 | | 0.423 | 0.417 | 0.417 |
| Obs. | 172,352 | 150,629 | 144,298 | 144,298 |
| B.Birth length | | | | |
| *O*3 | 0.052 | 0.010 | 0.032 | 0.024 |
|  | (0.048) | (0.056) | (0.067) | (0.068) |
| *N O* |  | -0.038 | -0.021 | -0.050 |
|  |  | (0.024) | (0.034) | (0.047) |
| *N O*2 |  |  |  | 0.104 |
|  |  |  |  | (0.115) |
| *r* 2 0.445 | | 0.432 | 0.424 | 0.424 |
| Obs. | 162,243 | 142,259 | 136,416 | 136,416 |

Notes: (1) Regressions are based on the benchmark model but include only *O*3, *N O* and *N O*2 in order to examine the correlation between the pollutants (2) The independent variables in Panel A and Panel B are birth weight and birth length separately. (3) Cluster robust standard errors at *grunnkrets* level in parentheses. (4) *** *p* 0.01, ** *p* 0.05, * *p* 0.1. (5) All pollutants are in

< < <

*µg* /*m*3, birth-weight in gram, birth length in millimeter.

*SO*2 concentrations. The concentration of *P M*2.5 is added last to column (6) due to the poor performance of the interpolation shown in Table [B1](#_bookmark25).

In Table [C4](#_bookmark36), columns (3)-(6), the estimation results are very imprecise because the monitoring values of *O*3 and *SO*2 concentrations are few, and their inclusion in the regression makes the sample size of the regression shrink dramatically. Column (3) is relatively less problematic, although the sample size is still cut in half. The magnitude and sign of the coefficients of *N O* are similar to the results of the baseline regression. More severe sample size decreases occur in columns (4)-(6), which have only a quarter of the sample size of the baseline regression, so I lack the power to accurately identify the effect. However, the sign of the coefficient on *N O* is still negative in these columns.

The coefficient of *N O*2 in column (4) of Table [C4](#_bookmark36) Panel B is even significantly positive, probably because there is a negative correlation between *N O* and *N O*2 in the process of reaching the photochemical equilibrium state in the samples of this group. Because *N O*2 is toxic anyway, this negative coefficient is not justified. And when the sample size is large enough, as in other columns, this negative correlation disappears, and the effect of *N O*2 is not significant. Notably, according to Table [C4](#_bookmark36), prenatal exposure to environmental *SO*2 appears to have a strong (but not significant due to small sample size) adverse effect on birth weight and length; after all, environmental *SO*2 is clearly a health issue in the few areas where it is monitored.

In addition to other pollutants, genetic diversity may be one of the potential confounding factors. According to the literature, air pollution has been associated with diabetes. Also, parental diabetes has been shown to have an effect on birth weight. Thus, parental diabetes is also a potential missed confounder. This phenomenon is known as genetic pleiotropy. If the only reason for the association of air pollution with birth outcomes is this genetic pleiotropy, *N O* would have an impact on birth outcomes once the parental history of diabetes is taken into account. Thanks to the detailed registration data, I can additionally control for parental diabetes history in column (7) of Table [C4](#_bookmark36). Here, parental diabetes history is a binary variable, indicating parental type I and type II diabetes. As we see in column (7), controlling for parental diabetes history does not affect the coefficients of *N O* in panels A and

Table C4: The effects of parental diabetes history and other ambient air pollutants in the 3rd. trimester on birth outcomes

| (1) | | (2) | (3) | (4) | (5) | (6) | (7) |
| --- | --- | --- | --- | --- | --- | --- | --- |
| baseline | | *P M*2.5 | *O*3 | *SO*2 | *SO*3&*O*2 | all | diabetes |
| A. Birth weight | |  |  |  |  |  |  |
| *N O* | -1.387∗∗ | -1.199∗ | -1.289 | -2.211 | -1.488 | -0.958 | -1.541∗∗ |
|  | (0.611) | (0.671) | (1.135) | (1.910) | (2.058) | (2.247) | (0.612) |
| *N O*2 | -0.259 | 0.520 | 0.917 | 5.324 | 4.857 | 4.881 | -0.220 |
|  | (1.762) | (1.906) | (2.808) | (4.982) | (5.285) | (5.400) | (1.772) |
| *P M*10 | 1.329 | 2.622 | 5.110∗ | 4.670 | 6.174 | 8.079 | 0.942 |
|  | (1.489) | (1.666) | (2.880) | (4.313) | (4.625) | (5.221) | (1.506) |
| *P M*2.5 |  | 0.589 |  |  |  | -7.799 |  |
|  |  | (2.117) |  |  |  | (12.976) |  |
| *O*3 |  |  | 0.017 |  | 1.184 | 1.809 |  |
|  |  |  | (1.530) |  | (2.780) | (3.039) |  |
| *SO*2 |  |  |  | -12.456 | -18.437 | -20.247 |  |
|  |  |  |  | (12.271) | (24.294) | (38.584) |  |
| *r* 2 0.464 | | 0.461 | 0.451 | 0.435 | 0.431 | 0.431 | 0.458 |
| Obs. | 225,239 | 203,845 | 115,434 | 55,829 | 52,194 | 51,985 | 239,561 |
| B.Birth length | | | | | | | |
| *N O* | -0.052∗ | -0.041 | -0.023 | -0.089 | -0.031 | 0.012 | -0.053∗ |
|  | (0.029) | (0.032) | (0.052) | (0.089) | (0.096) | (0.106) | (0.029) |
| *N O*2 | -0.023 | 0.027 | 0.027 | 0.284 | 0.197 | 0.217 | -0.032 |
|  | (0.080) | (0.086) | (0.129) | (0.218) | (0.236) | (0.242) | (0.081) |
| *P M*10 | -0.004 | 0.093 | 0.119 | 0.095 | 0.211 | 0.367 | -0.008 |
|  | (0.077) | (0.087) | (0.143) | (0.209) | (0.229) | (0.255) | (0.078) |
| *P M*2.5 |  | -0.106 |  |  |  | -0.669 |  |
| *O*3 |  | (0.095) | 0.047 |  | 0.219 | (0.619)  0.276∗ |  |
|  |  |  | (0.073) |  | (0.138) | (0.148) |  |
| *SO*2 |  |  |  | -0.145 | -0.648 | -0.856 |  |
|  |  |  |  | (0.591) | (1.054) | (1.780) |  |
| *r* 2 0.461 | | 0.459 | 0.452 | 0.440 | 0.437 | 0.437 | 0.462 |
| Obs. | 212,938 | 192,552 | 108,828 | 51,966 | 48,580 | 48,395 | 211,382 |

Notes: (1) Regressions are based on the benchmark model and include other types of pollutants in column 1-6 and parental diabetes history in column 7. (2) The independent variables in Panel A and Panel B are birth weight and birth length separately. (3) Cluster robust standard errors at *grunnkrets* level in parentheses. (4) *** *p* 0.01, ** *p* 0.05, * *p* 0.1. (5) All pollutants are in

< < <

*µg* /*m*3, birth-weight in gram, birth length in millimeter.

B. Therefore, I conclude that parental diabetes is not a channel through which *N O* affects birth weight and birth length.

In summary, based on the regression results in Table [C4](#_bookmark36), I do not find other pollutants such as *P M*2.5, *O*3, or *SO*2 as confounders of *N O*. Furthermore, in addition to *N O*, prenatal exposure to *SO*2 seems to have a negative effect on birth outcomes, whereas in the Norwegian environment, *P M* and *O*3 are at safe levels for newborns. I also find that parental diabetes is not a mechanism by which *N O* concentration affects birth weight and birth length. It is worth mentioning that designating *N O* and *N O*2 as the same pollutant, i.e., *N Ox* , may underestimate the negative health effects of *N O*, since it is *N O* and not *N O*2 that has adverse effects on the fetus, and the two pollutants are positively correlated, as mentioned in the literature review section.[61](#_bookmark38)

- 1. **Mothers relocating prior to childbirth**

Mothers moving between *grunnkrets* may cause serious measurement errors. If mothers move to a new area after childbirth, but within the same year, the address of mothers registered at the end of the year (*grunnkrets*) is not where they lived in the last trimester, that is, I incorrectly located where mothers lived before childbirth and also incorrectly measured the level of prenatal ambient air pollution exposure. On the other hand, moving before pregnancy may make the spatial fixation effect a “bad control” because pregnant women may move to avoid ambient air pollution.

In my data, I can observe in which *grunnkrets* (annual data) the mother lived and in which municipality the baby was born between 2000 and 2016. In the baseline analysis, I assume that the mother stays in the same place for the last trimester (and that the reason for choosing this place to live is not due to ambient air pollution), but in reality, it is common for mothers to move between *grunnkrets*. To investigate the above, I further divided the baseline sample into four groups based on the location of the mother and the place of birth of the infant, as shown in Table [C5](#_bookmark37):

Table C5: Sub-groups by maternal location in the year of childbirth

mothers delivered in another municipality yes no

| Group (1) move after delivery  4.2% | Group (2) move before delivery  10.3% |
| --- | --- |
| Group (3) no move, far hospital  24.9% | Group (4) no move, local hospital  60.6% |

mothers lived in another

yes

*grunnkrets* last year no

Notes: (1) Infants born between 2001-2016 are separated into 4 sub-groups (1)-(4) in the table. Those who were born in 2000 are not included because maternal location in 1999 are not traceable in my data. (2) The percentage following is the proportion each group accounts for. (3) Mothers who moved before delivery but chose to give birth in a different city also belongs to group (1), since I cannot distinguish them based on the data. (4) Because the mother’s location is updated annually, in groups (3) and (4), there may be mothers who move several times during the year and return to their original *grunnkrets* at the end of the year, although this is unlikely to happen in the last trimester before delivery.

- - - Group (1) moved immediately after delivery: The baby was born in a municipality other than the one where the mother lived in the year of delivery and the mother lived in two different *grunnkrets* in the year of delivery and the year before;
    - Group (2) moved before delivery: The baby was born in the same municipality where the mother lived in the year of delivery, but the mother lived in two different places in the year of delivery and the year before;
    - Group (3) delivered in hospitals far away: The mother did not move in the year of delivery, but the infant is born in another municipality;
    - Group (4) did not move: The mother did not move in the year of delivery, and the infant is also born in the same municipality where the mother lived.

Prenatal air pollution exposure for infants in group (1) may not have been measured correctly because the mothers likely moved after delivery (in the year of delivery). If so, the mothers’ registered address (*grunnkrets*) is not where they lived during the last trimester of pregnancy. Group 2 does not challenge my measurements as long as most mothers in the group moved during the first two trimesters rather than the last one. Mothers in group (3) gave birth in a different municipality than where they lived, but they did not move at all. This could have been self-selected or due to a lack of capacity at the local hospital. If the hospital is self-selected, there would be endogeneity problems.

− −

61I tried to identify the effect of *N Ox* on birth outcomes. The coefficients for *N Ox* are 0.771*g* for birth weight and 0.03*mm* for birth length (both significant at the 5% level, regression results not shown in the table), both of which are approximately half the coefficients in the baseline regression.

Mothers in group (4) lived in the same place in the year of delivery and before delivery and gave birth in the municipality where they lived, which is closer to the description of those who live stably in Norway.

Table C6: Robustness check: mothers moving between *grunnkrets*

| (1) | | | (2) | (3) | (4) | (5) |
| --- | --- | --- | --- | --- | --- | --- |
| Group 1 | | | Group 2 | Group 3 | Group 4 | Corrected |
| A. Birth weight | | |  |  |  |  |
| *N O* | 1.011 | | -3.354 | -2.083 | -0.157 | -1.267∗ |
|  | (3.949) | | (2.600) | (1.787) | (1.000) | (0.747) |
| *N O*2 | 2.025 | | -0.205 | -2.776 | -1.160 | -0.240 |
|  | (10.078) | | (7.466) | (4.430) | (2.901) | (2.230) |
| *P M*10 | 17.791 | | -7.858 | 9.236∗∗ | 3.119 | 2.364 |
|  | (12.918) | | (6.091) | (4.708) | (2.529) | (1.824) |
| *r* 2 0.695 | | | 0.620 | 0.561 | 0.509 | 0.469 |
| Obs. | 6,373 | | 15,140 | 37,542 | 90,416 | 143,675 |
| B.Birth length | | | | | | |
| *N O* | -0.122 | | -0.040 | -0.124 | -0.002 | -0.039 |
|  | (0.176) | | (0.164) | (0.076) | (0.045) | (0.036) |
| *N O*2 | 0.566 | | -0.611 | 0.109 | -0.142 | -0.097 |
|  | (0.429) | | (0.557) | (0.215) | (0.127) | (0.104) |
| *P M*10 | 0.885 | | -0.890 | 0.061 | 0.158 | 0.047 |
|  | (0.598) | | (0.705) | (0.193) | (0.125) | (0.100) |
| *r* 2 0.705 | | | 0.599 | 0.562 | 0.502 | 0.461 |
| Obs. | | 5,833 | 14,142 | 34,792 | 85,704 | 136,600 |
| C. Scenario | |  |  |  |  |  |
| *movem* | | yes | yes | no | no | - |
| *moveb* | | yes | no | yes | no | no |

Notes: (1) Regressions are based on the benchmark model for sub-samples labeled in panel C, where *mov em* indicates if the mothers moved between *grunnkrets* in the year and the year before delivery; *mov eb* indicates if the babies were born in the same municipality as where the mothers live in the year of delivery. (2) The independent variables in Panel A and Panel B are birth weight and birth length separately. (3) Cluster robust standard errors at *grunnkrets* level in parentheses. (4) *** *p* 0.01, ** *p* 0.05, * *p* 0.1. (5) All pollutants are in *µg* /*m*3, birth-weight in gram, birth length in millimeter.

< < <

Table [C6](#_bookmark39) reports the results of regressions by groups as discussed above. As usual, panels A and B of the table are for birth weight and length, respectively. Panel C indicates the subgroups of the sample involved in the regression. For example, in column (3) of Table [C6](#_bookmark39), the indicator *movem ye s* means that the mother lived in two different *grunnkrets* in the year of delivery and the year before; the indicator *moveb no* means that the infant was born in the same municipality where the mother lived in the year of delivery. Thus, column (2) corresponds to group (2) in Table [C5](#_bookmark37), i.e., mothers who moved prior to delivery.

=

=

Column (1) of Table [C6](#_bookmark39) reports the regression results for those whose prenatal exposure is wrongly measured (sample size of 1/40 of the baseline sample). The coefficients of pollutants makes no sense. Column (2) of Table [C6](#_bookmark39) indicates that infants of mothers who moved before delivery are more sensitive (although not significantly so) to ambient air pollution in the last trimester. This is explainable if the mothers in group (2) are those who are vulnerable to ambient air pollution. The mothers in column (3) of Table [C6](#_bookmark39) gave birth in a different municipality than where they lived, even though they themselves did not move at all in the year of delivery. Based on the coefficients in column (3), the babies of these mothers appear to be more vulnerable to ambient air pollution. This coincides with the self-selection of the maternity hospital. Perhaps vulnerable mothers and infants are the reason for choosing a distant hospital. In column (4), infants whose mothers did not move and gave birth in the same municipality are less affected by prenatal ambient air pollution exposure. This may be due to certain unobservable traits of these mothers, such as the fact that they may be long-time local residents of Norway.[62](#_bookmark40)

62Heterogeneity in the effect of prenatal ambient air pollution exposure on birth outcomes is discussed further in the next section.

The last column of Table [C6](#_bookmark39) excludes group (1) from the baseline sample. The prenatal exposure should thus be measured correctly. The results in this column are almost identical to the baseline regression results. In summary, neither the measurement error due to the mother’s move nor the self-selection of hospitals appeared to affect the baseline estimates after taking into account the birthplace of the infant.

#
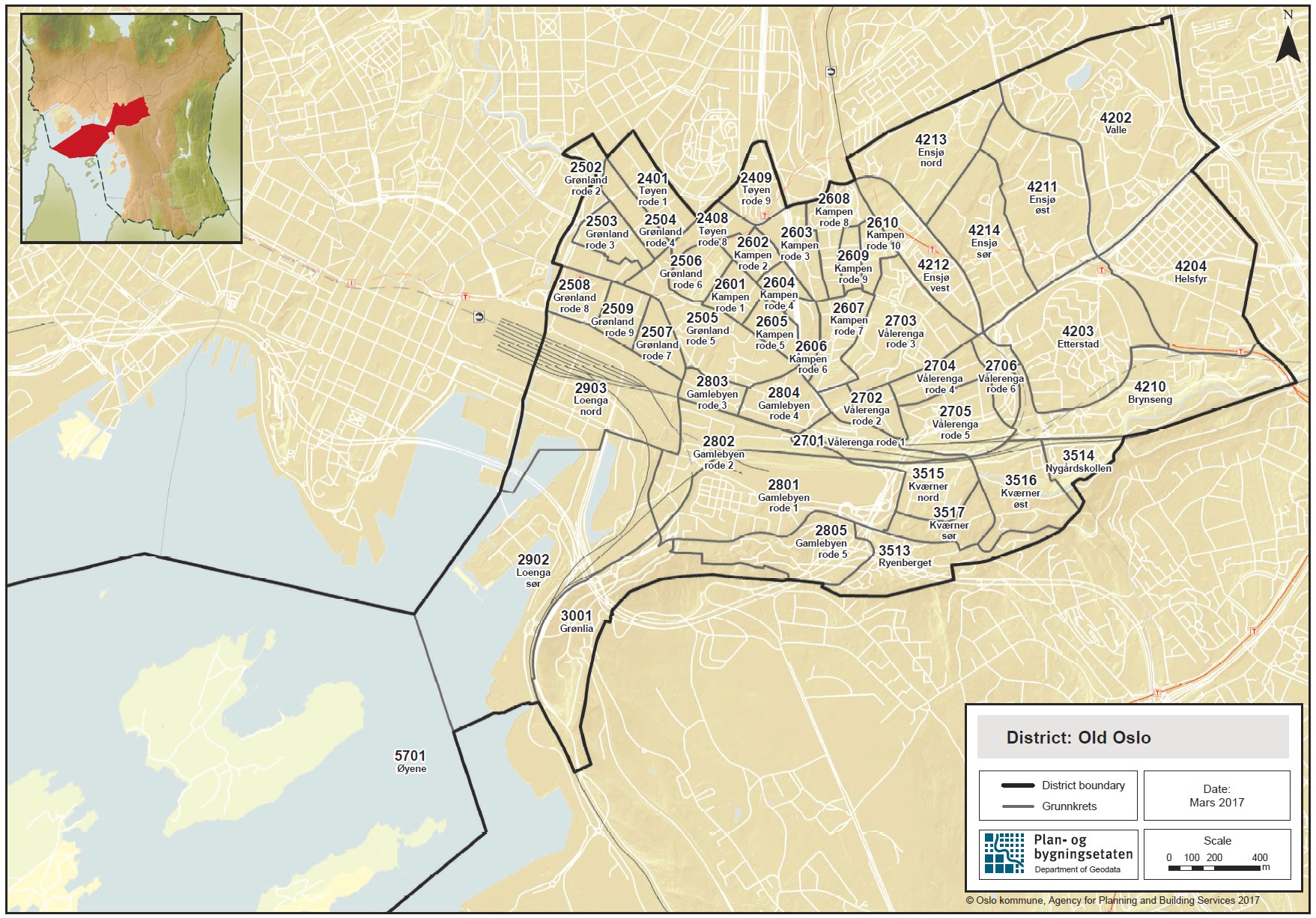
**Additional figures and tables**

Figure obtained from Oslo municipality: <https://www.oslo.kommune.no/statistikk/geografiske-inndelinger/>.

Figure D1: *Grunnkrets* in Old Oslo district of Oslo city


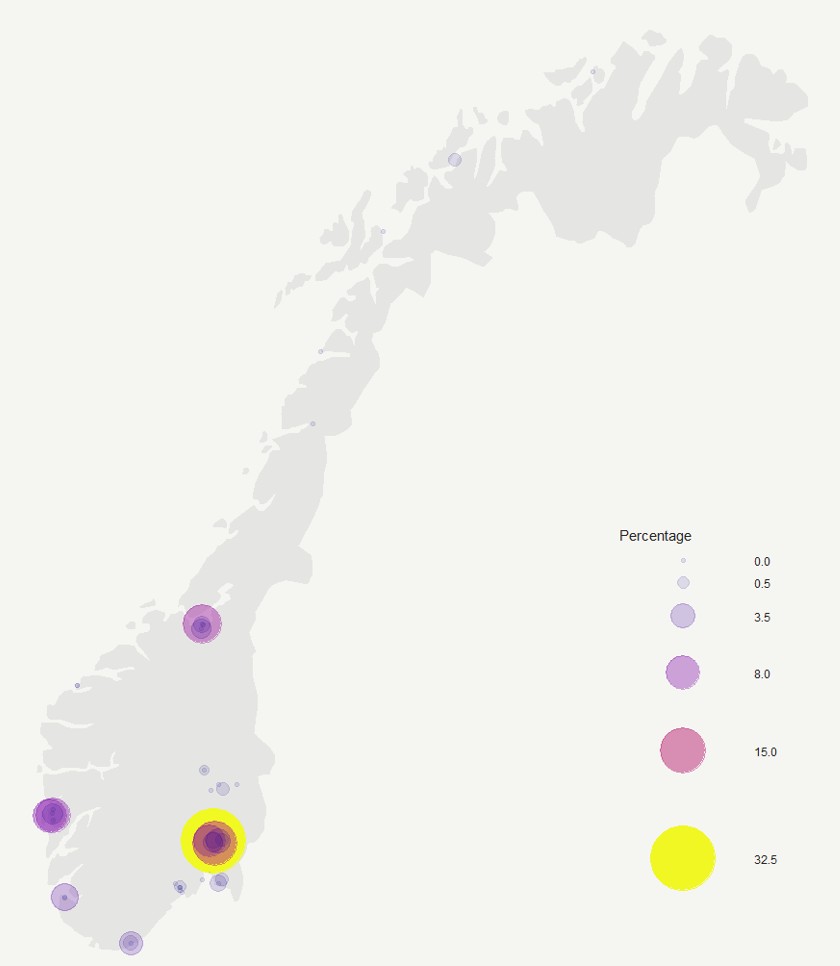


Figure D2: Percentage of weeks with high ambient *N O* concentration (> 110*µg* /*m*3) at the station level between 1999 and 2016


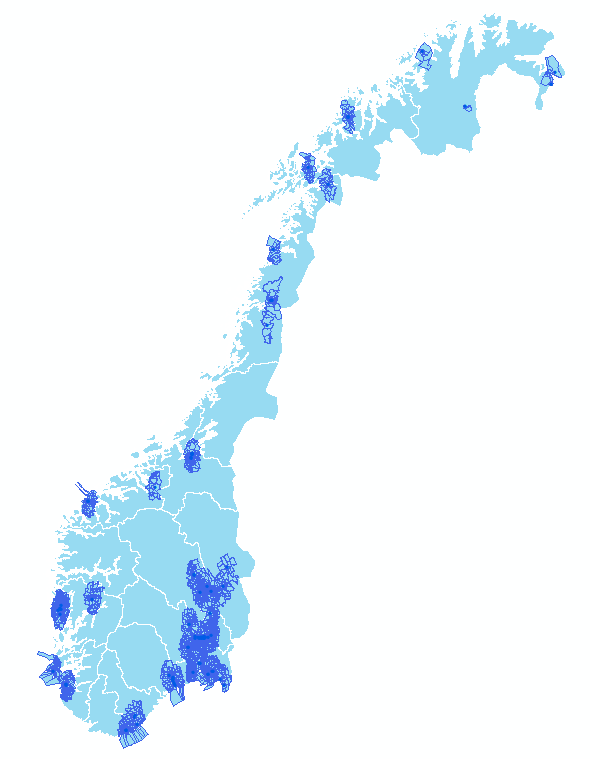


Figure D3: *Grunnkrets* with at least one air pollution monitoring stations within 20 miles


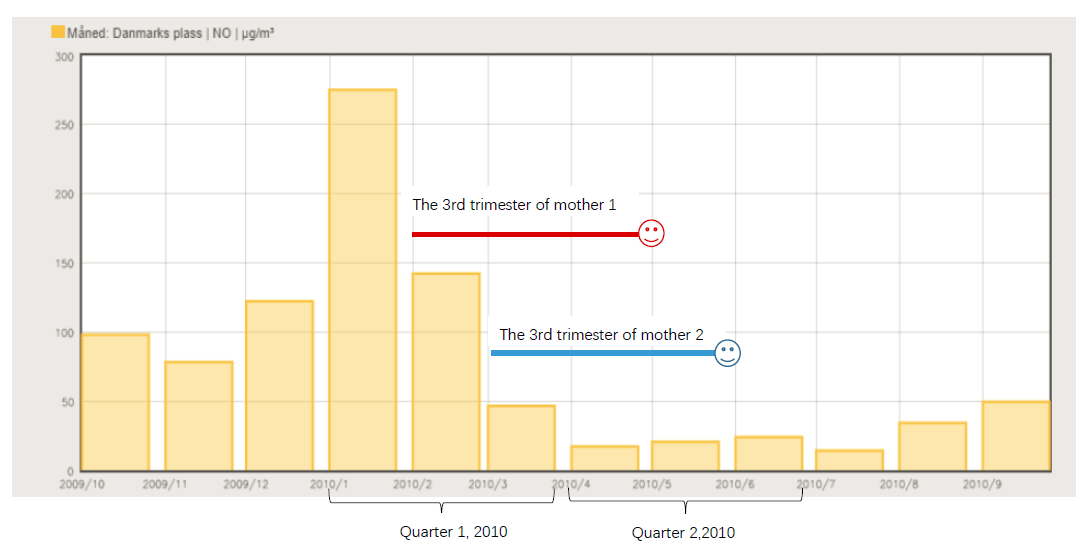


Figure D4: Two babies both born in the 2nd quarter of 2010, but have different prenatal exposure to *N O*


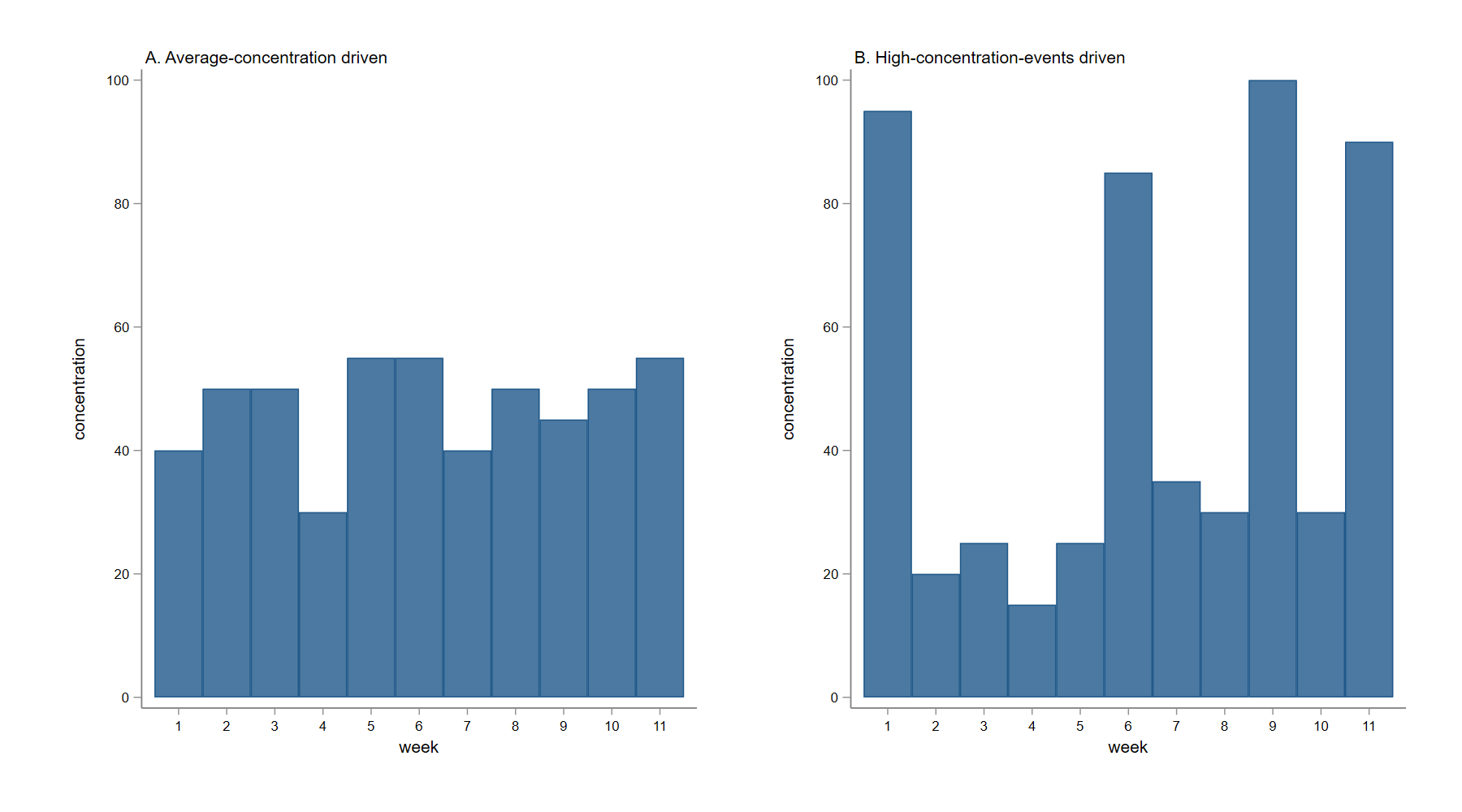


Figure D5: Given the same trimester-average concentration: relatively high concentrations in all weeks vs. occasion- ally very high concentration events.

Table D1: Air quality standards in different countries Pollutant averaging period EU USA China WHO

| annual | 40 | 100 | 40 | 40 |
| --- | --- | --- | --- | --- |
| *N O*2 daily | - | - | 80 | - |
| hourly | 200 | 188 | 200 | 200 |
| *P M*10 annual | 40 | - | 70 | 20 |
| daily | 50 | 150 | 150 | 50 |
| *P M*2.5 annual | 25 | 15 | 35 | 20 |
| daily | - | 35 | 75 | 25 |
| *SO*2 daily | - | 125 | 150 | 20 |
| hourly | 350 | 196 | 500 | - |
| *CO* daily | - | - | 4 | - |
| 8-hour | 10 | 10 | - | - |
| *O*3 8-hour | 120 | 137 | 160 | 100 |

Notes: (1) *CO* in *mg* /*m*3; all pollutants the other pollutants are measured in *µg* /*m*3. (2) There is no clear guideline for ambient *N O* in all these stan- dards. (3) There are different safety standards in the U.S. and the one in the is meant to “provide public health protection, including protecting the health of “sensitive" populations such as asthmatics, children, and the elderly”. (4) There are 2 classes of air quality standard in China and I adopt the one for residential zones (“2nd class”). (5) Data source: [European Com-](https://ec.europa.eu/environment/air/quality/standards.htm) [mission](https://ec.europa.eu/environment/air/quality/standards.htm), [European Environment Agency](https://www.eea.europa.eu/themes/data-and-maps/figures/air-quality-standards-under-the), [U.S. Environmental Protection](https://www.epa.gov/criteria-air-pollutants/naaqs-table#2) [Agency](https://www.epa.gov/criteria-air-pollutants/naaqs-table#2), [Ministry of Ecology and Environment People’s Republic of China](http://www.mee.gov.cn/ywgz/fgbz/bz/bzwb/dqhjbh/dqhjzlbz/201203/t20120302_224165.shtml), [World Health Organization](https://www.who.int/news-room/fact-sheets/detail/ambient-(outdoor)-air-quality-and-health#%3A%7E%3Atext%3DThe%202005%20WHO%20Air%20quality%2Crelated%20deaths%20by%20around%2015%25),

Table D2: Month of childbirth in the sample and the rest part of the population

|  | Baseline | sample |  | Population | uncovered |
| --- | --- | --- | --- | --- | --- |
| Month | Freq. | Percent |  | Freq. | Percent |
| 1 | 38,134 | 8.21 |  | 45,213 | 8.30 |
| 2 | 35,973 | 7.75 |  | 42,481 | 7.80 |
| 3 | 39,380 | 8.48 |  | 46,224 | 8.48 |
| 4 | 39,699 | 8.55 |  | 46,769 | 8.58 |
| 5 | 42,001 | 9.05 |  | 47,633 | 8.74 |
| 6 | 41,450 | 8.93 |  | 46,587 | 8.55 |
| 7 | 43,027 | 9.27 |  | 49,247 | 9.04 |
| 8 | 40,780 | 8.78 |  | 48,254 | 8.86 |
| 9 | 37,867 | 8.16 |  | 46,993 | 8.63 |
| 10 | 37,745 | 8.13 |  | 44,271 | 8.13 |
| 11 | 34,500 | 7.43 |  | 40,722 | 7.47 |
| 12 | 33,748 | 7.27 |  | 40,418 | 7.42 |

Total 464,304 544,812

Table[D2](#_bookmark41) shows the distribution of birth months in my sample. The month of delivery is evenly distributed, with slightly more babies being born in the summer months. Some parents may prefer to have their babies in early summer (May-July), either to avoid the summer heat or to take parental leave between summer vacations (so they can enjoy a "double summer vacation"). Information such as the fact that babies born in the summer get more vitamin D and are therefore healthier (which is essential in high latitude countries such as Norway) may also be at play here. I also compared the demographics of parents with “summer babies” and those without “summer babies” and found no significant differences.

Table D3: Benchmark regression with standard errors clustered at different levels

| (1) | | (2) | (3) | (4) | (5) | (6) | (7) | (8) | (9) |
| --- | --- | --- | --- | --- | --- | --- | --- | --- | --- |
| cluster: p | | muni | family | q-g | m-p | q-p | s | m-s | q-s |
| A.Birth weight | |  |  |  |  |  |  |  |  |
| *N O* | -1.387∗ | -1.387∗∗∗ | -1.387∗∗ | -1.387∗∗ | -1.387∗∗ | -1.387∗∗ | -1.387∗∗ | -1.387∗∗ | -1.387∗∗ |
|  | (0.068) | (0.008) | (0.035) | (0.027) | (0.026) | (0.033) | (0.011) | (0.011) | (0.017) |
| *N O*2 | -0.259 | -0.259 | -0.259 | -0.259 | -0.259 | -0.259 | -0.259 | -0.259 | -0.259 |
|  | (0.902) | (0.841) | (0.881) | (0.891) | (0.881) | (0.890) | (0.876) | (0.876) | (0.887) |
| *P M*10 | 1.329 | 1.329 | 1.329 | 1.329 | 1.329 | 1.329 | 1.329 | 1.329 | 1.329 |
|  | (0.455) | (0.244) | (0.412) | (0.318) | (0.413) | (0.315) | (0.364) | (0.421) | (0.331) |
| B.Birth length | | | | | | | | | |
| *N O* | -0.052 | -0.052∗∗∗ | -0.052∗ | -0.052∗ | -0.052∗ | -0.052∗ | -0.052∗∗ | -0.052∗∗ | -0.052∗∗ |
|  | (0.141) | (0.000) | (0.087) | (0.065) | (0.089) | (0.069) | (0.035) | (0.041) | (0.023) |
| *N O*2 | -0.023 | -0.023 | -0.023 | -0.023 | -0.023 | -0.023 | -0.023 | -0.023 | -0.023 |
|  | (0.816) | (0.710) | (0.784) | (0.770) | (0.779) | (0.769) | (0.804) | (0.800) | (0.792) |
| *P M*10 | -0.004 | -0.004 | -0.004 | -0.004 | -0.004 | -0.004 | -0.004 | -0.004 | -0.004 |
|  | (0.967) | (0.944) | (0.962) | (0.956) | (0.961) | (0.956) | (0.952) | (0.954) | (0.943) |

Notes: (1) The standard errors are clustered at level: p(postcode), muni(municipality), family(children of the same mother), q-g(calendar quarter and *grunnkrets*), m-p(calendar month and postcode), q-p(calendar quarter and postcode), s(nearest monitoring station), m-s(calendar month and nearest monitoring station) and q-s(calendar quarter and nearest monitoring station). (2) The independent variables in Panel A and Panel B are birth weight and birth length separately. (3) *** *p* < 0.01, **

*p* < 0.05, * *p* < 0.1. (4) All pollutants are in *µg* /*m*3, birth-weight in gram, birth length in millimeter.

Table D4: Air pollution exposure during the whole pregnancy

|  | | birth weight |  |  |  | birth length |  |
| --- | --- | --- | --- | --- | --- | --- | --- |
| trimester | all | same | stay |  | all | same | stay |
| *N O* | 0.958 | 1.181 | 0.884 | 0.022 | | 0.017 | 0.049 |
|  | (0.773) | (0.970) | (1.093) | (0.036) | | (0.046) | (0.052) |
| 1*st N O*2 | -1.568 | -2.520 | -2.226 | -0.065 | | -0.072 | -0.133 |
|  | (2.058) | (2.582) | (2.765) | (0.097) | | (0.127) | (0.125) |
| *P M*10 | 1.151 | 1.826 | -0.396 | 0.105 | | 0.088 | 0.108 |
|  | (1.882) | (2.192) | (2.600) | (0.091) | | (0.103) | (0.120) |
| *N O* | 0.699 | 0.803 | 0.063 | -0.046 | | -0.091 | -0.001 |
|  | (1.268) | (1.585) | (1.676) | (0.060) | | (0.073) | (0.078) |
| 2*nd N O*2 | 2.587 | 1.747 | 0.331 | 0.238∗ | | 0.203 | 0.158 |
|  | (2.866) | (3.571) | (3.673) | (0.138) | | (0.166) | (0.169) |
| *P M*10 | 1.225 | 4.367 | 2.223 | -0.048 | | 0.154 | -0.083 |
|  | (3.182) | (3.731) | (4.267) | (0.146) | | (0.171) | (0.189) |
| *N O* | -1.010 | -0.777 | -0.692 | -0.063 | | -0.066 | -0.048 |
|  | (0.858) | (1.052) | (1.102) | (0.040) | | (0.049) | (0.052) |
| 3*r d N O*2 | 0.786 | -0.003 | -0.566 | 0.071 | | -0.024 | 0.023 |
|  | (2.168) | (2.718) | (2.801) | (0.102) | | (0.133) | (0.127) |
| *P M*10 | 1.610 | 3.475 | 3.984 | -0.019 | | 0.101 | 0.077 |
|  | (1.952) | (2.402) | (2.657) | (0.104) | | (0.136) | (0.125) |
| *r* 2 0.464 | | 0.469 | 0.502 | 0.462 | | 0.461 | 0.501 |

Obs. 211,935 135,535 135,714 200,292 128,855 127,816

Notes: (1)The three trimesters are regressed in one model, not separately. (2) *al l* means all observations in the working data is used; *same* means mothers lived in the same munici- palities in the delivery year as where the children were born, *st a y* keeps only those whose mothers resided in the same *grunnkrets* in the delivery year and the year before delivery. (3) Cluster robust standard errors at the *grunnkrets* level in parentheses. (4) *** *p* 0.001, ** *p* 0.01, * *p* 0.05. (5) All pollutants are in *µg* /*m*3, birth-weight in gram, birth length in millimeter.

In Table [D4](#_bookmark42), I include average ambient air pollution levels and weather conditions throughout the three trimester of pregnancy in the baseline regression model. The samples in columns (3) and (5) are the baseline sample (“all”), while the infants in columns (4) and (6) (“same”) are born in the same city as their mother’s residence in the year of delivery, as a robustness check (as discussed in Section (2)).

< <

<

When air pollution and weather conditions in the first two quarters are included, the *N O* coefficients in the last three months remain very similar to the baseline regressions of birth weight and birth length. Although most of the coefficients in Table [D4](#_bookmark42) are not significant at the 10% level, the coefficients on *N O*2 and *P M*10 are less significant, i.e., the magnitude of the effect is smaller, and the standard error is larger. Based on the findings in Table [D4](#_bookmark42), I included only the last three months of air pollution levels and weather conditions in the baseline analysis.

Table D5: Excluding mothers exposed to high-level ambient air pollution in 3rd. trimester

|  | (1) |  | (2) |  | (3) |  | | (4) |  | (5) (6) | |  | (7) (8) | |
| --- | --- | --- | --- | --- | --- | --- | --- | --- | --- | --- | --- | --- | --- | --- |
|  |  | *N O* |  |  |  | *N O*2 | |  |  | *P M*10 | |  | *N O*2 & *P M*10 | |
|  | < 99*p*. |  | < 95*p*. |  | < 99*p*. |  | | < 95*p*. |  | < 99*p*. < 95*p*. | |  | < 99*p*. < 95*p*. | |
| A.Birth weight | | | | | | | | | | | | | | |
| *N O* | -1.896∗∗ | 0.756 | | -2.342∗∗∗ | | | -2.717∗∗∗ | | -1.240∗ | | -1.448 | -2.298∗∗∗ | | -2.012 |
|  | (0.784) | (1.151) | | (0.693) | | | (0.954) | | (0.707) | | (0.920) | (0.785) | | (1.224) |
| *N O*2 | 0.765 | -0.445 | | 1.750 | | | 0.693 | | 0.472 | | 1.626 | 2.933 | | 1.139 |
|  | (1.886) | (2.118) | | (1.906) | | | (2.551) | | (2.017) | | (2.545) | (2.207) | | (3.336) |
| *P M*10 | 1.140 | 0.537 | | 1.716 | | | 0.745 | | 0.871 | | -0.287 | 0.592 | | 1.429 |
|  | (1.620) | (1.763) | | (2.079) | | | (2.995) | | (1.620) | | (1.863) | (2.274) | | (3.583) |
| *r* 2 0.467 | | 0.468 | | 0.464 | | 0.468 | | | 0.467 | | 0.470 | 0.466 | | 0.470 |
| Obs. | 205,170 | 165,555 | | 206,770 | | 156,122 | | | 206,417 | | 163,289 | 190,339 | | 127,154 |
| B.Birth length | | | | | | | | | | | | | | |
| *N O* | -0.053 | 0.082 | | -0.085∗∗ | | -0.099∗∗ | | | -0.053 | | -0.012 | -0.082∗∗ | | -0.055 |
|  | (0.038) | (0.055) | | (0.033) | | (0.045) | | | (0.034) | | (0.043) | (0.037) | | (0.054) |
| *N O*2 | 0.007 | -0.056 | | 0.068 | | 0.008 | | | 0.014 | | 0.020 | 0.124 | | 0.006 |
|  | (0.087) | (0.101) | | (0.084) | | (0.111) | | | (0.092) | | (0.117) | (0.097) | | (0.145) |
| *P M*10 | -0.054 | -0.096 | | -0.046 | | -0.117 | | | -0.017 | | -0.128 | -0.078 | | -0.086 |
|  | (0.083) | (0.094) | | (0.099) | | (0.137) | | | (0.083) | | (0.097) | (0.107) | | (0.163) |
| *r* 2 0.463 | | 0.466 | | 0.462 | | 0.466 | | | 0.464 | | 0.467 | 0.465 | | 0.470 |
| Obs. | 194,486 | 157,291 | | 195,830 | | 148,232 | | | 195,498 | | 155,014 | 180,669 | | 121,101 |

Notes: (1) Regressions are based on the benchmark regression model in babies whose mother were not exposed to high-level ambient air pollution. Here 99*p*. and 95*p*. means that the weekly maternal exposure to certain pollutants in the third trimester is all below 99th and 95th percentile. (2) Cluster robust standard errors at *grunnkrets* level in parentheses,

(3) *** *p* < 0.01, ** *p* < 0.05, * *p* < 0.1. (5) All pollutants in *µg* /*m*3, birth-weight in gram, birth length in millimeter.

The regressions in Table [D5](#_bookmark43) are for certain environmental contaminants in the subsample where mothers were not exposed to any high levels of environmental contamination events (weekly mean concentrations >99*t h*/95*t h* percentile) in the last trimester. For example, weekly prenatal environmental *N O* exposures in the last trimester (11 weeks total) in column (1) were below the 99*t h* percentile. Columns (1) and (2) are copied from columns (1) and (5) in table (6 for comparison purposes. We can find that the coefficient on *N O* is no longer negative when all 11 weeks of prenatal exposure to *N O* in the last trimester are below the 95*t h* percentile, and the exclusion of high levels of environmental *N O*2 and *P M*10 pollution events does not affect the sign of the coefficient on *N O*. This means that it is the high level of *N O*, not *N O*2 or *P M*10, that reduces the birth outcome.
